# Thoracolumbar epidural stimulation effects on bladder and bowel function in uninjured and chronic transected anesthetized rats

**DOI:** 10.1038/s41598-022-06011-2

**Published:** 2022-02-08

**Authors:** Robert F. Hoey, Daniel Medina-Aguiñaga, Fahmi Khalifa, Beatrice Ugiliweneza, Dengzhi Wang, Sharon Zdunowski, Jason Fell, Ahmed Naglah, Ayman S. El-Baz, April N. Herrity, Susan J. Harkema, Charles H. Hubscher

**Affiliations:** 1 Department of Anatomical Sciences and Neurobiology, University of Louisville School of Medicine, Louisville, KY USA; 2 Bioengineering Department, University of Louisville J. B. Speed School of Engineering, Louisville, KY USA; 3 Department of Neurological Surgery, University of Louisville School of Medicine, Louisville, KY USA; 4 Kentucky Spinal Cord Injury Research Center, University of Louisville, Louisville, KY USA; 5 Physical Medicine and Rehabilitation Department, MetroHealth Rehabilitation Institute of Ohio, Cleveland, OH USA; 6 Department of Health Management and Systems Science, School of Public Health and Information Science, University of Louisville, Louisville, KY USA

**Keywords:** Spinal cord diseases, Urological manifestations

## Abstract

Pre-clinical studies have shown that spinal cord epidural stimulation (scES) at the level of pelvic and pudendal nerve inputs/outputs (L5-S1) alters storage and/or emptying functions of both the bladder and bowel. The current mapping experiments were conducted to investigate scES efficacy at the level of hypogastric nerve inputs/outputs (T13-L2) in male and female rats under urethane anesthesia. As found with L5-S1 scES, T13-L2 scES at select frequencies and intensities of stimulation produced an increase in inter-contraction interval (ICI) in non-injured female rats but a short-latency void in chronic T9 transected rats, as well as reduced rectal activity in all groups. However, the detrusor pressure during the lengthened ICI (i.e., urinary hold) remained at a low pressure and was not elevated as seen with L5-S1 scES, an effect that’s critical for translation to the clinic as high fill pressures can damage the kidneys. Furthermore, T13-L2 scES was shown to stimulate voiding post-transection by increasing bladder activity while also directly inhibiting the external urethral sphincter, a pattern necessary to overcome detrusor-sphincter dyssynergia. Additionally, select scES parameters at T13-L2 also increased distal colon activity in all groups. Together, the current findings suggest that optimization of scES for bladder and bowel will likely require multiple electrode cohorts at different locations that target circuitries coordinating sympathetic, parasympathetic and somatic outputs.

## Introduction

Among the myriad of complications that follow spinal cord injury (SCI), bladder and bowel dysfunctions are rated by the SCI population as top priority issues impacting quality of life^1,2^. Additionally, bladder and bowel health are among the most common reasons for re-hospitalization after SCI and are a major source of morbidity. After injury, deficits develop in both storage and emptying dynamics (for example, neurogenic detrusor overactivity, NDO; detrusor sphincter dyssynergia; impaired motility in the bowel), leading to incontinence and retention^3^.

Treatment approaches for daily management involve pharmacological and/or physical interventions to directly address symptoms and maintain day-to-day health^4^. The gold standard and first-line treatment include emptying the bladder via a catheter (clean intermittent catheterization) as well as digital stimulation for defecation, with or without use of suppositories or enemas. Oral medication (anticholinergics, β3 agonist, and α-blockers) represents the second line of treatment. Persons with persistent NDO and/or incontinence are candidates for the use of botulinum neurotoxin (Botox) to improve capacity and reduce low volume bladder contractions from NDO. Botox injections into the bladder wall produces temporary benefits^5–7^ that may become less effective long term^7^ and it suppresses contractility necessary for emptying^7^. Those requiring further intervention may benefit from novel stimulation focused on neuromodulation including^8^: electrical stimulation of the tibial^9–12^, saphenous^13,14^, pudendal nerves^15,16^, and the dorsal genital nerve^17,18^; electrical stimulation of the sacral nerves^19,20^; sacral anterior root stimulation (SARS) combined with posterior root rhizotomy^21–23^; and the recently described laproscopic neuroprosthesis implantation^24^. Surgical methods, such as bladder augmentation or colostomy^4^, are reserved for those patients in which all other treatment strategies have failed to produce sufficient benefit.

Recent stimulation techniques have targeted the spinal cord rather than peripheral nerves to influence peripheral function. Spinal cord epidural stimulation (scES) utilizes an electrode array that is implanted on the surface of the dura mater and has been shown to improve function in people with impairments due to neurogenic bladder^25–30^. Although initially targeted for standing and stepping^31^, many other benefits have been uncovered, including multiple autonomic functions, suggesting that scES can improve off-target function via interaction with spinal circuits that extend across numerous levels of the spinal cord. These effects are consistent with a recent study utilizing transcutaneous stimulation to improve bladder function^32^. Several preliminary scES clinical case reports showing benefits of directly targeting spinal circuitries mediating bladder and bowel function prompted mapping studies for identification of spatial specificity and optimal parameters for achieving storage and emptying for both lower urinary tract (LUT) and anorectal function in rodent models that have been used extensively for pre-clinical studies of SCI^33^.

Previous data from our laboratory obtained from acute terminal studies in non-injured and chronic-transected male and female urethane-anesthetized adult rats indicated numerous scES-induced effects on bladder and bowel functions within the lumbosacral region of the spinal cord (L5-S1; level of inputs/outputs of the pelvic and pudendal nerves)^33^. For the current experiments, the thoracolumbar region of the spinal cord was targeted (T13-L2; level of inputs/outputs of the hypogastric nerve). The T13-L2 scES results are discussed and considered relative to the scES findings from lumbosacral level mapping.

## Results

**Figure 1:**
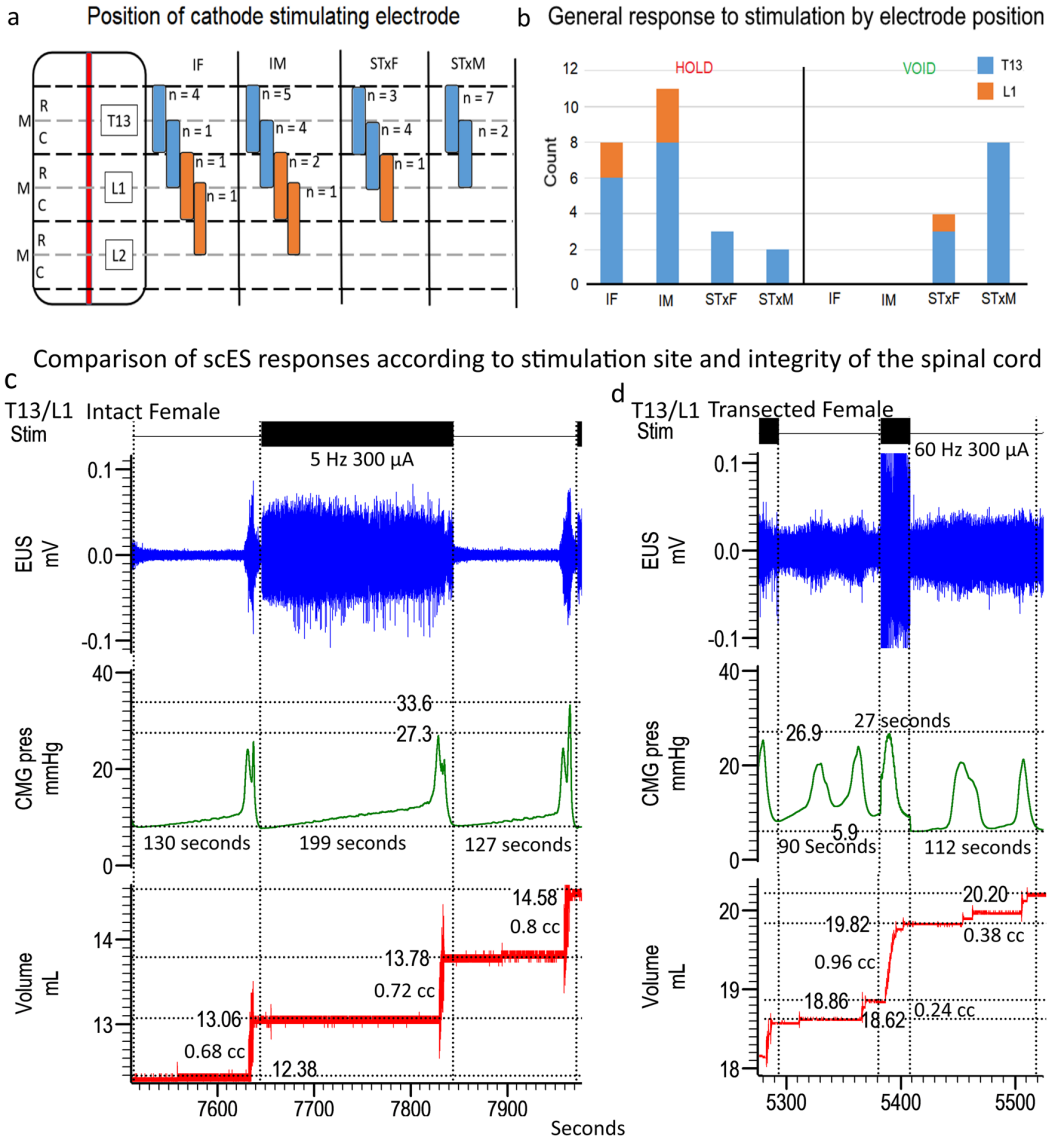
The placement of the cathode stimulating electrode on the spinal cord for each animal is shown (**a**) with the gross response type for each group (**b**, either hold or void responses), and traces of both the intact (**c**) and transected (**d**) responses. “Hold” was defined as inhibition of bladder activity seen as complete blockade of bladder contractions leading to OI or a lengthening of the ICI (**c**). A “void” is a bladder contraction occurring shortly after stimulation is turned on (within 30 s) and much earlier than the previous ICI would predict (**d**). The majority of electrode placements included either all or some portion of the T13 level (blue bars; IF 6/8; IM 8/11; STxF 6/7; STxM 10/10). Some placements were caudal to T13 and were either all or some portion of L1 (orange). As shown in (**b**), the placement (either T13 or L1) of the electrode did not seem to be responsible for either a hold or void response. Because the intact animals never had voiding responses to stimulation, but the transected animals did, the void response to scES is most likely due to the injury and subsequent plasticity. Data traces showing bladder responses to high intensity scES (300–500 µA) in intact female rats at T13/L1 (**c**) and transected female rats at T13/L1 (**d**). High intensity scES at T13/L1 prolongs the filling phase. As seen in example (**c**), scES at T13/L1 causes a 53% increase in the time until a void occurs (green trace). During scES the CMG trace is smooth and rises at a slope that is similar to the non-stimulated periods prior to and after. The low pressure and lack of substantial EUS EMG activity suggests that the prolonged time until void is due to bladder inhibition (and increased compliance). The transected female response with an immediate void (**d**) caused an increase in CMG pressure and a substantial increase in voided volume. Data from panel (**c**) has an EUS response that is not representative of the overall mean effect of the group.

Data sets collected from intact females (IF; n = 7/11), intact males (IM; n = 12/12), transected females (STxF; n = 8/9), and transected males (STxM; n = 9/12) were analyzed according to sex, injury, and stimulation parameters. Note that the “Methods” section “Definitions” contains a description of each measure. A summary figure illustrating the post-mapping experiment dissection finding of electrode placement and general response results is provided in Fig. 1a,b.

For data reduction, per our previously published study^33^, mapping parameters were grouped according to low (5 and 10 Hz) versus high (30, 45, and 60 Hz) frequency and below versus above visualized movement stimulus intensity threshold (VisMvt; 50, 75, 100 µA and 150 and 300 µA, respectively). These four groupings presented as four quadrants (Q1–Q4) on heat maps for each functional outcome and analysis using the following designations: (Q1) low frequency, below VisMvt; (Q2) high frequency, below VisMvt; (Q3) high frequency; above VisMvt; and (Q4) low frequency, above VisMvt. In addition, between and within group differences were assessed with and without epidural stimulation (Stim On and Stim Off conditions, respectively).

### Bladder function results

The average of five baseline void volumes per rat collected after consistent fill-void cycle patterns were established (filling at a pump speed of 0.25 cc/min) were similar (p > 0.05) for intact females (0.61 cc, SEM = 0.09), intact males (0.52 cc, SEM = 0.05), transected females (0.52 cc, SEM = 0.11), and transected males (0.47 cc, SEM = 0.02). As established previously^33^, a subset of SCI rats (100% of males and 37.5% of females) displayed an overflow incontinence (OI) phenotype whereby upon reaching capacity, small drops of urine would leak upon small detrusor pressure increases rather than expelled as an intermittent stream with a reflex bladder contraction.

When scES was applied to the T13-L2 region (“Stim On”), there were changes in bladder function (Fig. 1c,d for examples of responses, Supplemental Table 1) consistent with a storage effect in intact animals (Fig. 1c) and a micturition response in transected animals (Fig. 1d). Note that during the extended hold time, the detrusor pressure remained low.

**Figure 2:**
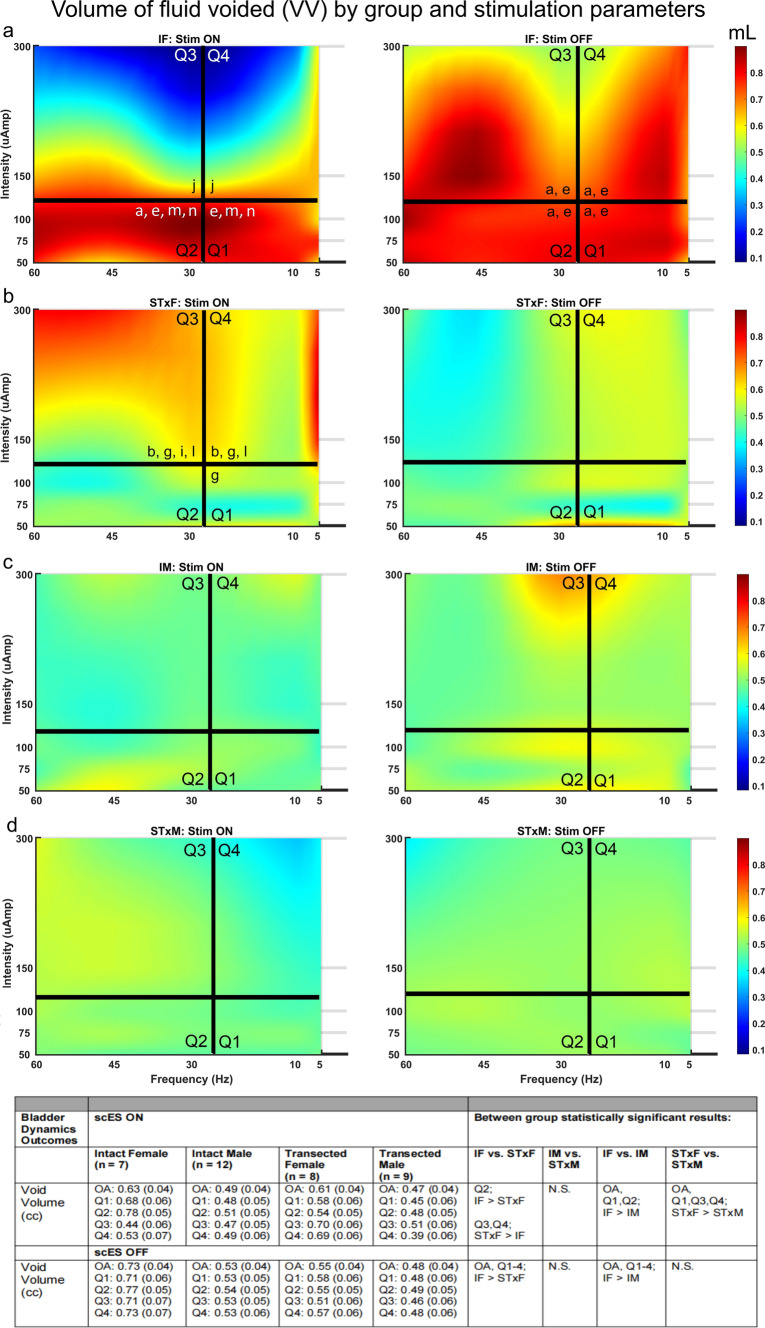
Summary heat maps (headings **a**-**d**) showing Voided Volume (VV), with (left column, Stim ON) and without (right column, Stim Off) scES at T13/L1. The heat map is colored so that volume is represented by either hot (orange, red) or cold (blue) colors based on high or low volume voided, respectively. The number of animals included in these data were as follows: IF, n = 7; STxF, n = 8; IM, n = 12; STxM, n = 9. Statistical notation for between group differences: (a) IF > STxF; (b) STxF > IF; (c) IM > STxM; (d) STxM > IM; (e) IF > IM; (f) IM > IF; (g) STxF > STxM; (h) STxM > STxF. Statistical notation for within group differences: (i) On > Off; (j) Off > On; (k) > Q1; (l) > Q2; (m) > Q3; (n) > Q4. Overall, the female groups (IF and STxF) had greater VV than male groups in both stimulation conditions (**e**,**g**). Within the IF group (top row), high intensity stimulation (Q3, Q4) caused a significant reduction in VV (**j**, left pane, blue section) with a compensatory void afterward (right pane, dark red in Q3, Q4). The opposite response occurred in the STxF group with Q3 stimulation causing increased VV with a short-latency void (second row, left pane, red section). This quadrant was significantly greater than IF (**b**), than STxM (**g**), and than Stim Off (**i**). A similar, but non-significant pattern was observed in males (IM and STxM) which is interpreted in the discussion.

**Figure 3:**
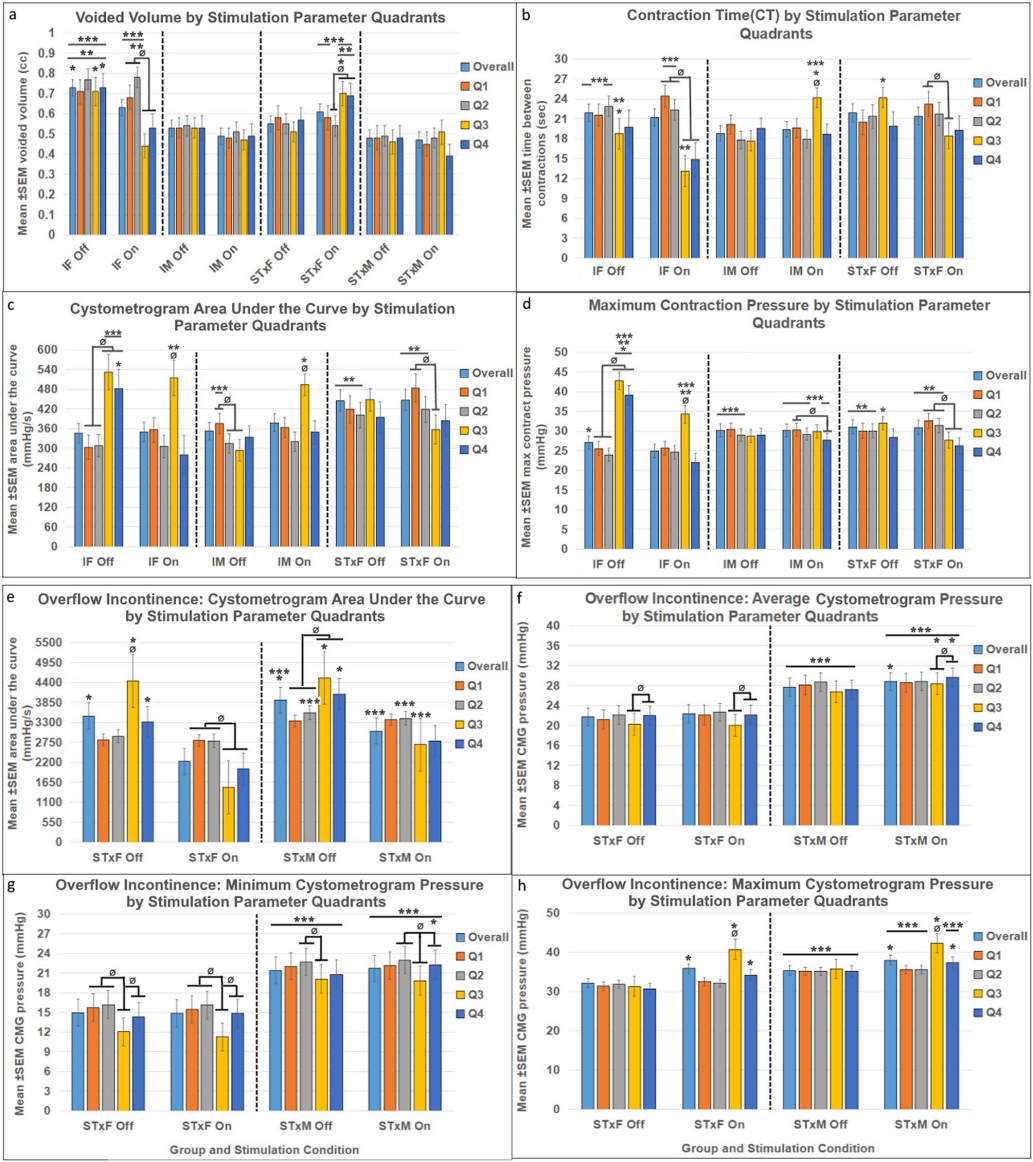
Bladder responses to T13/L2 scES in animals with cycling bladders (**a**–**d**) and animals with overflow incontinence (**e**–**h**). Because a subset of animals did not have cycling bladders it was not possible to include them in all types of analysis (CT, CMG AUC, Max pressure) and therefore they were analyzed separately. Group numbers were as follows: Volume (IF n = 7; STxF n = 8; IM n = 12; STxM n = 9); CT, CMG AUC, Max pressure (IF n = 7; STxF n = 5; IM n = 12); all Overflow Incontinence measures (STxF n = 3; STxM n = 9). Statistical notation: Ø = within group difference, * = within On vs. Off difference, ** = between group difference intact vs transected, *** = between group difference male vs. female (within injury groups, I.e. intact male vs. Intact female). Error bars represent ± SEM.

Intact females displayed reduced void volumes (VV) with Stim On at sufficient intensity (150–300 µA; see Q3, Q4 in Figs. 2a, 3a). In contrast, VV increased after chronic spinal cord transection in female rats during high intensity stimulation (150–300 µA; see Q3, Q4 in Figs. 2b, 3a), with Stim On > Off in Q3. The within group differences (Fig. 3a) are only present with scES. Similar effects were not found in males and neither intact nor transected males showed significant changes in VV from stimulation (Figs. 2c,d, 3a).

Both intact and transected males had consistently lower VV than females (Fig. 3a) with Stim On, and intact males also had lower VV than females with Stim Off. The contraction time (CT; duration of voiding contractions) was also significantly different between groups with intact females having longer ICI’s than intact male rats (Fig. 3b) except with high frequency/above VisMvt scES where intact males had greater ICI (Fig. 3b).

Effects of T13/L2 scES were also found for: the cystometrogram area under the curve (AUC, Fig. 3c), and mean maximum contraction pressure (Fig. 3d). Intact females had intensity dependent increases in AUC and max pressure with Stim Off (Q3,4 > Q1,2) as well as elevations in both measure with Stim On (Q3). Results from intact males show a similar effect with Stim On for AUC (Q3, Fig. 3c) but not for Stim Off or maximum pressure. These data correspond to the reduced voided volume in these groups (Figs. 2c, 3a). Finally, transected females had reduced AUC and maximum pressure with Stim On (Q1,2 > Q3,4) which correspond to greater voided volume.

### Non-contractile bladder results

The proportion of animals in OI per group is consistent with our previous study^33^, whereby the bladder pressure recordings indicate oscillations around fluid being pumped in and leaking out at capacity. This pressure data could not be quantified in the same way as the other animals and so was analyzed separately for: AUC, mean pressure, maximum pressure, and minimum pressure (Fig. 3e–h). T13-L2 scES induced voiding in transected animals, regardless of OI or cycling activity, as shown by the significantly greater AUC when stimulation intensity was below VisMvt (Q1, 2 > Q3, 4) in both male and female rats. Additionally, when stimulation intensity was high (Q3 and 4) maximum pressure and mean pressure were significantly greater, respectively for both transected males and females.

### EUS EMG results

**Figure 4:**
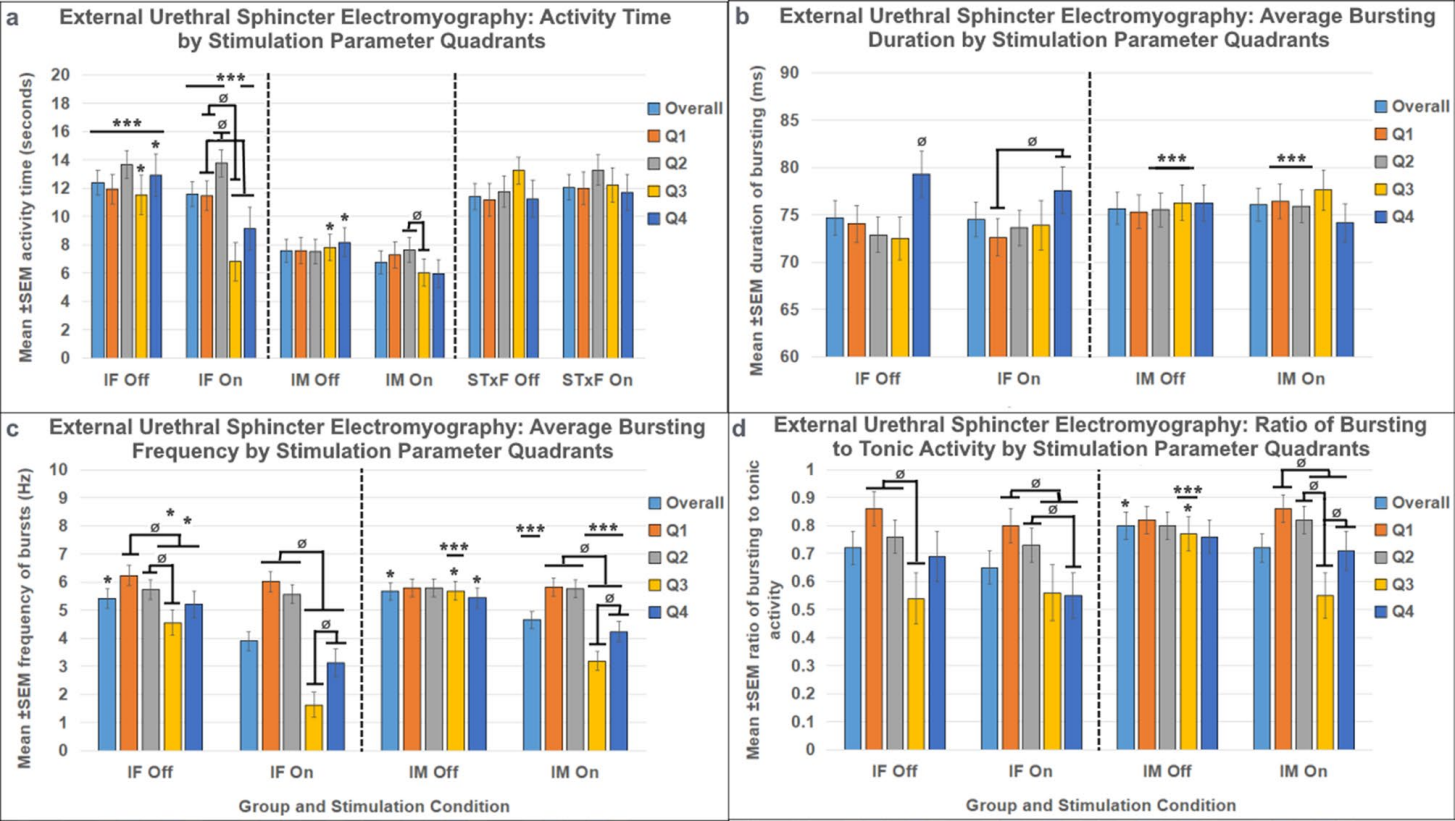
External urethral sphincter electromyography responses to T13/L2 scES. Mean activity time (**a**), mean bursting duration (**b**), mean bursting frequency (**c**), and mean ratio of bursting to tonic activity time (**d**) data are presented. The y-axis of (**b**) is truncated to better show the statistical differences. Group numbers were as follows: Mean activity time (IF n = 7; STxF n = 8; IM n = 12); mean bursting time, mean bursting frequency, and ratio of tonic to bursting activity (IF n = 7; IM n = 12). Statistical notation: Ø = within group difference, * = within On vs. Off difference, ** = between group difference intact vs transected, *** = between group difference male vs. female (within injury groups, I.e. intact male vs. Intact female). Error bars represent ± SEM.

T13-L2 scES produced numerous differences in EMG related variables (Fig. 4, Supplemental Table 1): EMG total activity time (Fig. 4a); mean bursting time (Fig. 4b); mean bursting frequency (Fig. 4c); and bursting On:Off ratio (Fig. 4d). Consistent with previous data^33^, transected males and females did not have EUS bursting activity after chronic SCI. Data from animals that did have bursting activity indicate sex differences in EMG results, with intact females having significantly longer EUS EMG activity time and with intact males having significantly greater outcomes related to EUS bursting (burst duration, frequency, and ratio). High intensity scES stimulation that is above the VisMvt threshold yielded reduced EUS activity in both males and females, as well as a long-lasting aftereffect that reduces EUS activity in the Stim Off period directly following stimulation.

### Bowel function results

**Figure 5:**
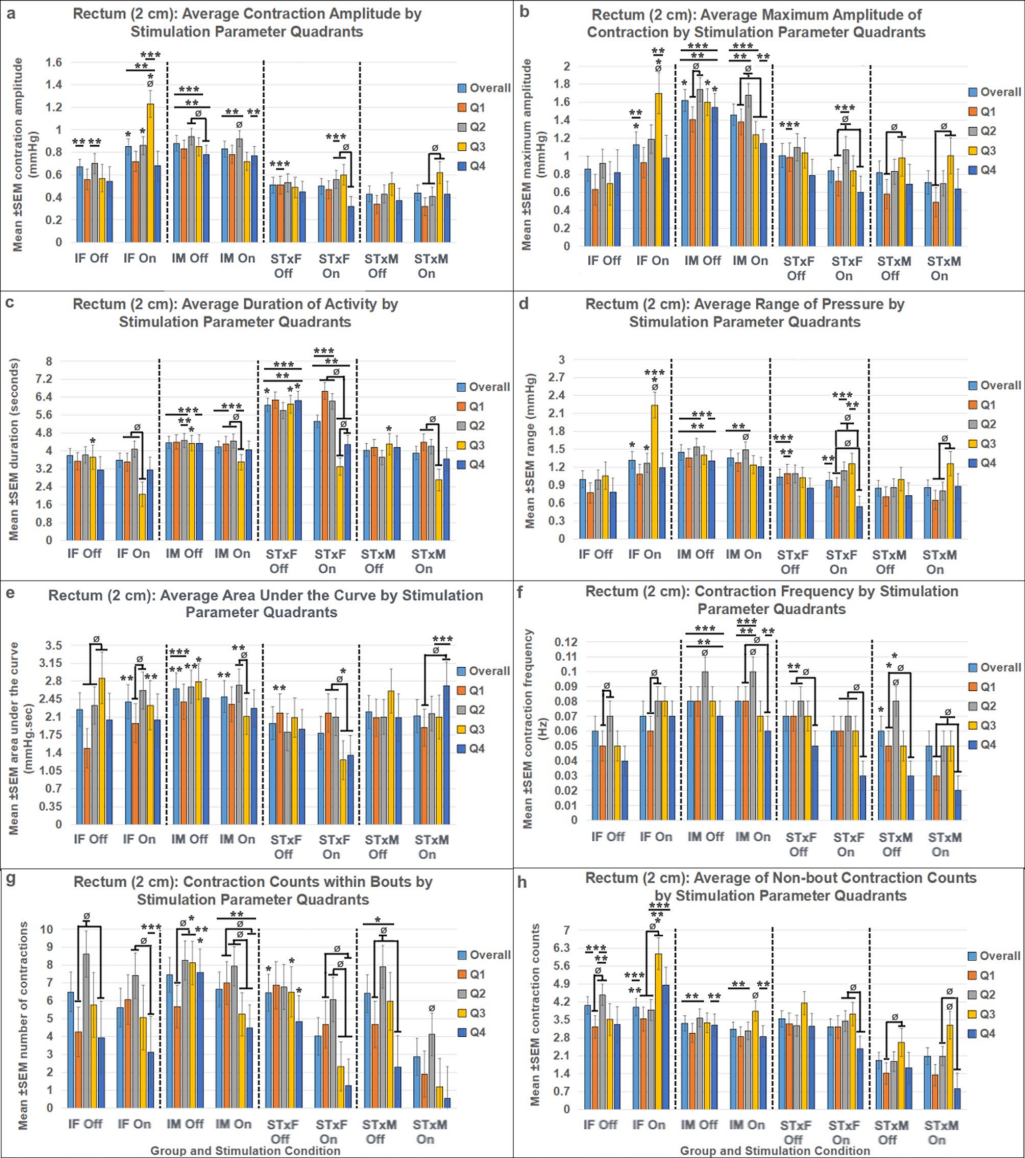
Responses in the rectum (2 cm past the anal verge) to T13/L2 scES. Outcome measures were: mean contraction amplitude (**a**), mean maximum amplitude (**b**), mean duration of activity (**c**), mean range of pressure (**d**), mean area under the curve (AUC, **e**), mean contraction frequency (**f**), mean winthin bout contraction counts (**g**), and mean non-bout contraction count (**h**). Group numbers were the same for all outcome measures (IF n = 7; STxF n = 8; IM n = 12; STxM n = 9). Statistical notation: Ø = within group difference, * = within On vs. Off difference, ** = between group difference intact vs transected, *** = between group difference male vs. female (within injury groups, I.e. intact male vs. Intact female). Error bars represent ± SEM.

**Figure 6:**
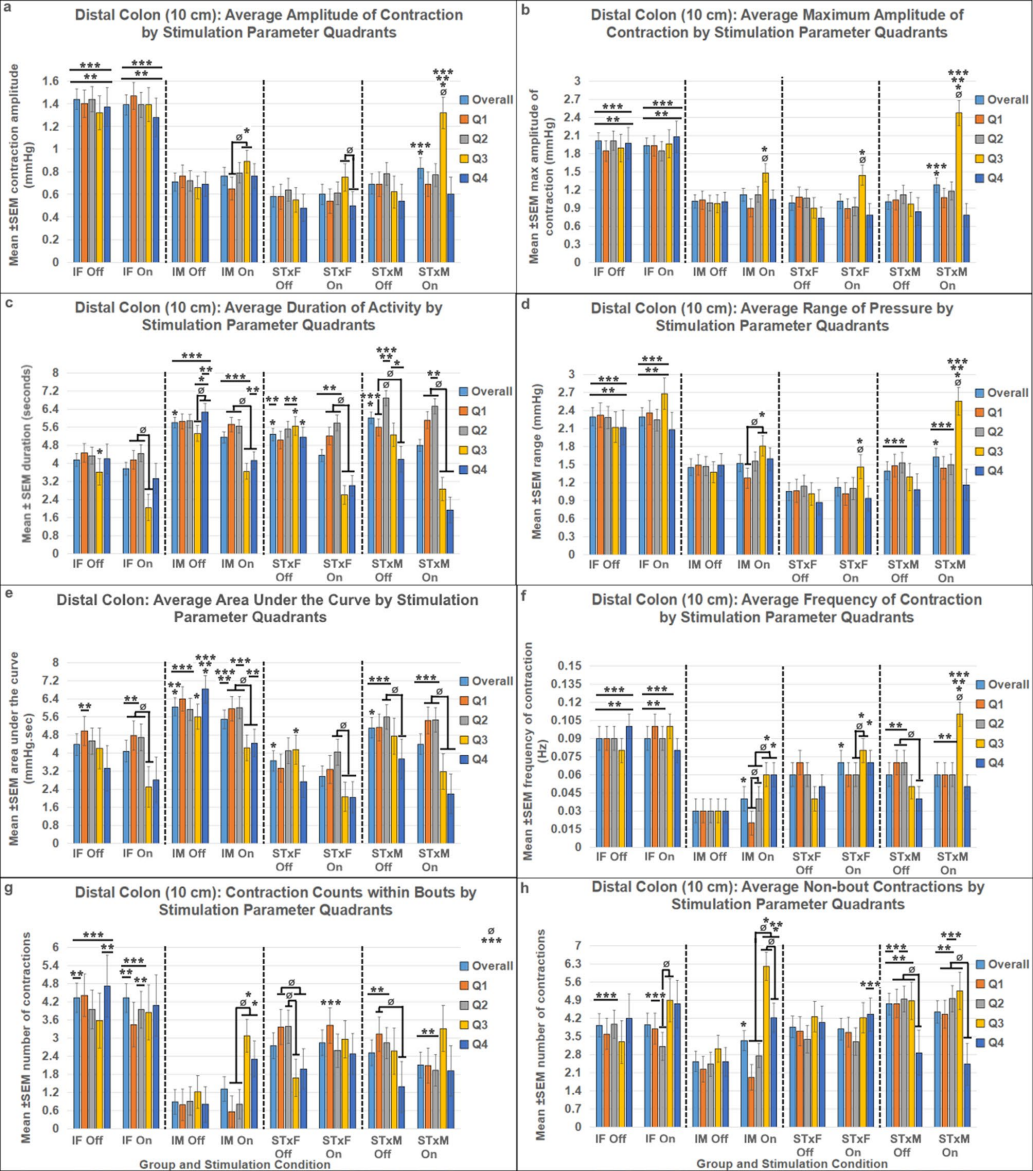
Responses in the distal colon (10 cm past the anal verge) to T13/L2 scES. Outcome measures were: mean contraction amplitude (**a**), mean maximum amplitude (**b**), mean duration of activity (**c**), mean range of pressure (**d**), mean area under the curve (AUC, **e**), mean contraction frequency (**f**), mean winthin bout contraction counts (**g**), and mean non-bout contraction count (**h**). Group numbers were the same in all outcome measures (IF n = 7; STxF n = 8; IM n = 12; STxM n = 9). Statistical notation: Ø = within group difference, * = within On vs. Off difference, ** = between group difference intact vs transected, *** = between group difference male vs. female (within injury groups, I.e. intact male vs. Intact female). Error bars represent ± SEM.

**Figure 7:**
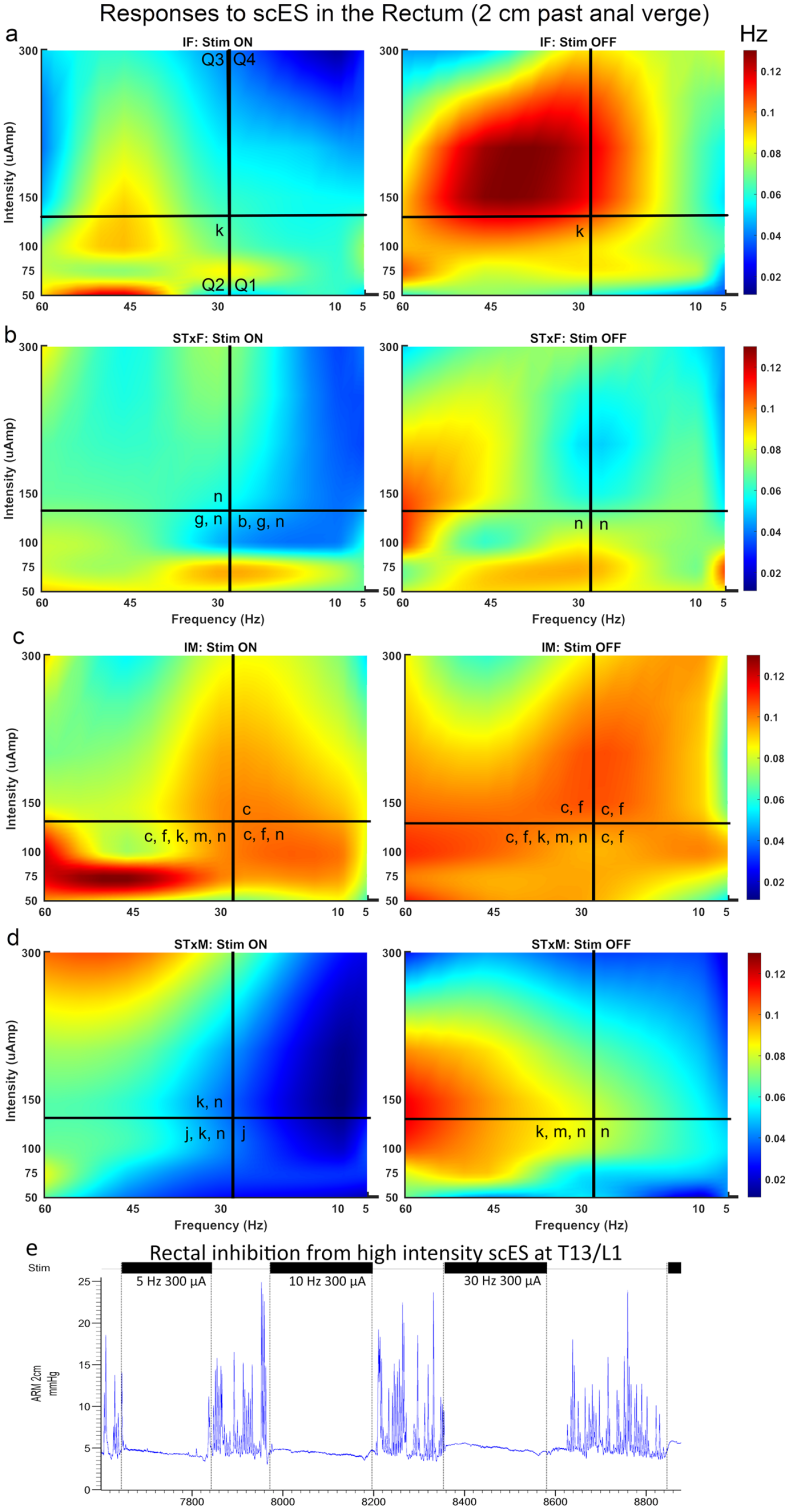
Summary heat map showing contraction frequency, with and without scES at T13/L2, in the rectum (2 cm from the anal verge) and a representative trace. During scES, the rectum (**a**–**d**) had a lower contraction frequency than when Stim was Off (note hot colors in the right pane of **a**–**d**). The trace (**e**) clearly shows inhibition of rectal contractions when stimulation is being applied (black bars) at high intensity and recovers when stimulation is turned off. The number of animals included in these data were as follows: IF, n = 7; STxF, n = 8; IM, n = 12; STxM, n = 9. Statistical notation for between group differences: (a) IF > STxF; (b) STxF > IF; (c) IM > STxM; (d) STxM > IM; (e) IF > IM; (f) IM > IF; (g) STxF > STxM; (h) STxM > STxF. Statistical notation for within group differences: (i) On > Off; (j) Off > On; (k) > Q1; (l) > Q2; (m) > Q3; (n) > Q4.

**Figure 8:**
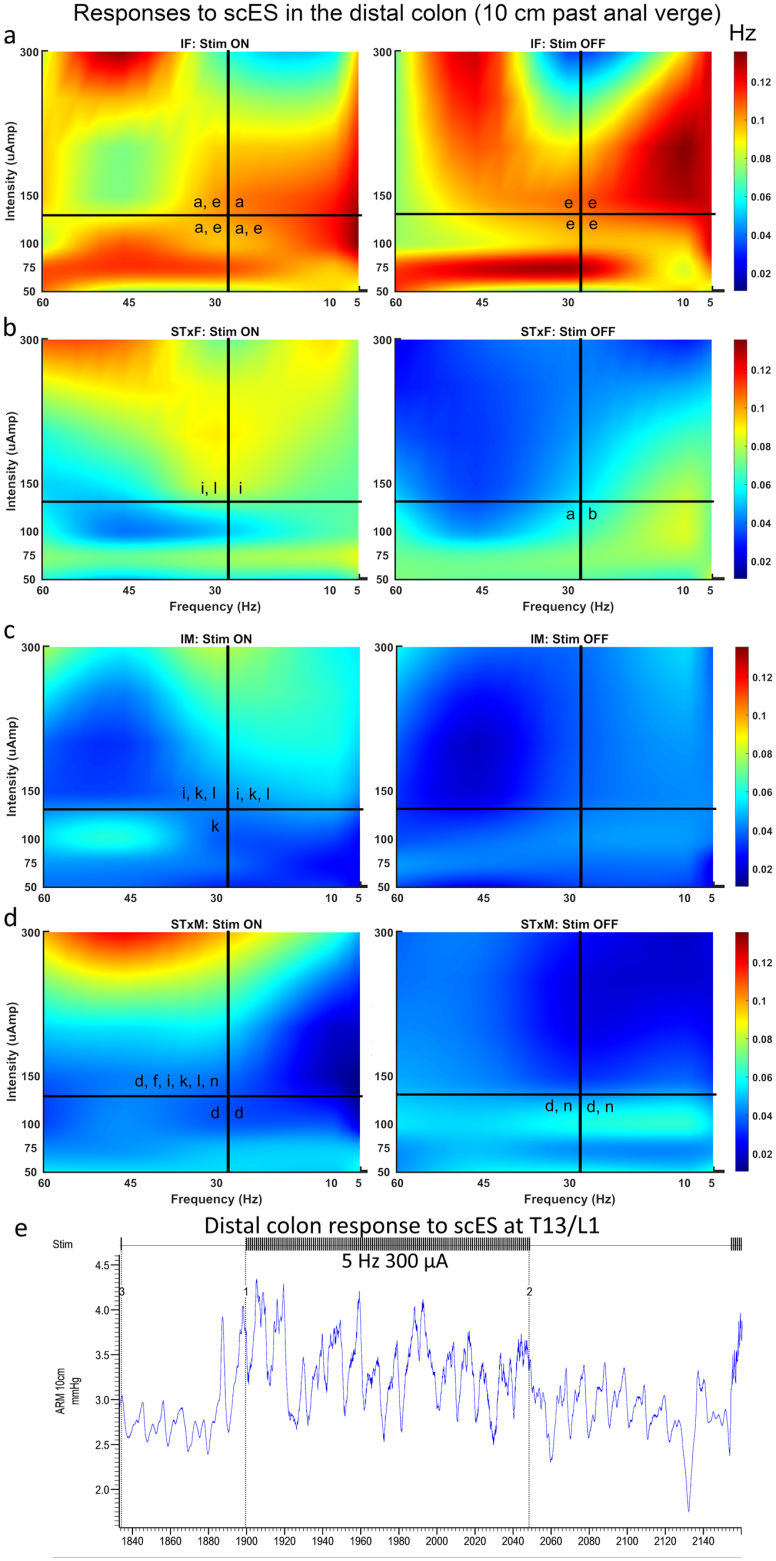
Summary heat map showing contraction frequency, with and without scES at T13/L1, in the distal colon (10 cm from the anal verge) and a representative trace. The distal colon had higher contraction frequency during scES shown by hot colors on left during Stim On and cool colors on right during Stim Off. The number of animals included in these data were as follows: IF, n = 7; STxF, n = 8; IM, n = 12; STxM, n = 9. Statistical notation for between group differences: (a) IF > STxF; (b) STxF > IF; (c) IM > STxM; (d) STxM > IM; (e) IF > IM; (f) IM > IF; (g) STxF > STxM; (h) STxM > STxF. Statistical notation for within group differences: (i) On > Off; (j) Off > On; (k) > Q1; (l) > Q2; (m) > Q3; (n) > Q4.

Rectal (2 cm beyond the anal verge, Fig. 5, Supplemental Table 2) and distal colon (10 cm beyond the anal verge, Fig. 6, Supplemental Table 3) function were differentially influenced by T13-L2 scES. Outcome measures quantified for rectal and distal colon function included: mean amplitude (Figs. 5a, 6a); maximum amplitude (Figs. 5b, 6b); mean duration (Figs. 5c, 6c); mean range (Figs. 5d, 6d); mean AUC (Figs. 5e, 6e); mean contraction frequency (Figs. 5f, 6f, 7, 8); contraction count within bouts (Figs. 5g, 6g); contraction count non-bout (Figs. 5h, 6h). Bowel pressure data was collected using Millar sensors via a plastic membrane near the tip of the probe that transduce pressure (in mmHg) when impinged upon by muscle contractions in the tissue. Rectal activity was inhibited by T13-L2 scES across all intact and transected groups. Overall, group differences in rectal function (2 cm ARM results) did not change based on stimulation condition indicating that those differences were a result of sex and injury dynamics rather than the response to stimulation. For example, IM is always greater than STxM and STxF is always greater than STxM regardless of stimulation condition, although greater inhibition did occur in some comparisons when scES was applied. When stimulation is Off, STxF is nearly always greater than IF; but when scES is applied, the relationship flips, and most outcome measures (except duration and frequency) show that IF has greater activity than STxF. This finding indicates that T13-L2 scES is more strongly inhibiting the STxF than the IF animals. This interpretation is supported by within comparisons showing the most common relationship found was that Stim Off was greater than Stim On (Fig. 5), indicating that application of scES to T13-L2 reduces rectal activity across groups. In contrast, T13-L2 level scES on distal colon function (10 cm ARM, Fig. 6, Supplemental Table 3) induced increased colonic activity, with nearly all groups showing greater contraction frequency (Fig. 8) and mean amplitude with Stim On than Off (STxF, IM, STxM). Although collected, external anal sphincter electromyographic data are not presented here.

### VisMvt results

Consistent with the previous experiment^33^ T13-L2 scES was found to cause muscular contractions in numerous areas of the body. Interestingly, even though the electrode placement in the current experiment was rostral by several spinal levels (T13-L2 vs. L5-S1) the distribution of VisMvt was similar. Specifically, the areas of movement (bilateral) were as follows: axial muscles along the spinal column, flanks (caudal to the bottom rib and rostral to knee), hips, leg (knee joint, ankle joint, toes), genital/perineal area (bulbospongionsus, scrotum, and penis in males), anal region (sphincter and adjacent area), and base of the tail. Stimulation was considered a “complete” response when all motor areas were activated by stimulation. The threshold for movements were determined after the mapping procedure. The movement threshold was considered the lowest stimulus intensity that produced a visible contraction in any of the regions (above). Mean movement thresholds (SEM), not significantly different (one-way ANOVA, p = 0.7), were as follows: intact female = 129.83 µA (16.57), intact male = 180.60 µA (18.42), transected female = 123.25 µA (8.73), and transected male = 117.89 µA (9.59).

## Discussion

The current findings demonstrate that scES applied to the thoracolumbar (T13-L2) region of the spinal cord, acutely at 6 weeks post-injury, has many similar effects to scES of the lumbosacral region (L5-S1) at the same time point^33^. Specifically, rats with an intact spinal cord responded to a similar stimulation intensity with a hold (or an inhibition of voiding and reduced volume), and groups having a chronic T9 spinal cord transection responded to stimulation with a short-latency void and increased volume when stimulation intensity was above VisMvt. Furthermore, bowel responses were also consistent with previous L5-S1 results showing an intensity-dependent inhibition of rectal contractions across all groups. However, some important differences were found. For the intact groups, the hold dynamics of T13-L2 stimulation was a low-pressure filling phase rather than the high-pressure filling with OI that was characteristic of L5-S1 stimulation^33^, a clinically relevant difference with respect to safety, as elevated pressures within the LUT may cause reflux and damage to the kidneys. For bowel, T13-L2 scES also produced an increase in distal colon contraction frequency, indicating different locations/parameters for targeting storage and movement of feces.

### T13-L2 scES responses

Epidural stimulation of the T13-L2 region of the spinal cord, at 6 weeks post-injury, induced changes in the activity of both the bladder and bowel with high (above-VisMvt) intensity stimulation that included regions below (ankle-L4-5, toes-L3-L5, genital/perineal-L6-S2) the dermatomes supplied by this level of the spinal cord (T13-L2)^34^. That scES is causing muscle contractions in regions that are innervated by other levels of the cord suggests that scES is interacting with spinal circuits that can influence the activity of other regions of the cord. Although identifying the exact targets for these effects is beyond the scope of the current experiments (an important avenue of inquiry for future studies), the outcome data when examined as a whole suggests that the electric field necessary for generating responses is likely stimulating a variety of regions. Potential targets not only include the sympathetic outflow at the level of stimulation, but also intra-spinal networks related to the coordination of micturition-related events (between the spinal cord gray matter of T13-L2 and L5-S1) and activation of descending circuits within the white matter.

The duplication of an injury-dependent response to stimulation (hold-intact versus void-transected) with T13-L2 scES suggests that chronic SCI induces plasticity in the control of LUT circuits that can be activated by stimulation at multiple loci (spinal level of sympathetics and parasympathetics). Furthermore, the presence of a hold response (Fig. 1b) in some transected animals (typically seen in intact animals) suggests that local circuitry is involved and that some circuit reorganization is occurring allowing for the void response (Fig. 1b,d). Indeed, intact animals having no deviation in response (Fig. 1b) suggests that injury-induced plasticity is responsible for the change from “hold” to a “void” response in transected animals as the void response does not occur in uninjured animals when using similar scES parameters. This finding is consistent with the literature that shows a novel pathway after SCI that can directly activate the bladder after neuroplastic changes^35^. Furthermore, this data is consistent with previous work showing L5-S1 stimulation causing a similar effect after chronic transection^33^. Taken together, these studies suggest that scES can activate spinal circuits related to micturition at both the T13-L2 and L5-S1 levels.

### scES-induced lower urinary tract effects

The current experiment has provided more evidence that LUT function can be directly influenced by scES. Previous results from scES at L5-S1 showed a bladder response that quickly rose in pressure during filling and throughout the filling phase^33^. This type of filling is unsafe and conflicts with the low pressure fill recommended guidelines of the International Continence Society^36^, as it can cause damage to both the detrusor muscle (via distension and trauma to the muscle itself) and the kidneys (via reflux through the ureter). Alternatively, scES of T13-L2 produces an inhibition of the bladder that allows for low-pressure filling most likely via activation of sympathetic pathways^37–40^ that promote bladder relaxation and compliance. Previous studies have shown that blockade of sympathetic circuits increases compliance in cats^37,39^, dogs^38^, and rats^40^. In addition, it is also possible that scES directly activates the spinal GABAergic system that has been shown to cause bladder inhibition due to pudendal stimulation^41^. Thus, scES targeting the maintenance of continence may be more advantageous at the level of T13-L2 for increasing capacity with low-pressure filling and increased compliance than L6-S1.

Following chronic transection (6 weeks post-injury), the response to scES at T13-L2 is a short-latency void (Fig. 1d, green trace) instead of the hold response (Fig. 1c, green trace) that occurs in intact animals. This effect also occurs in response to L5-S1 scES^33^ which suggests a fundamental change in the LUT response to scES after injury. Often after SCI, bladder emptying is a challenge that requires physical intervention, typically catheterization, due to strong activation of the EUS at the same time as the detrusor contraction (detrusor-sphincter dyssynergia, DSD)^3^. The current results show in transected animals that a void response from T13-L2 stimulation is characterized by increased VV, increased bladder pressure, and reduced EUS EMG. This finding is significant because stimulation was able to incite a more typical voiding pattern after chronic transection (contracting bladder with relaxing EUS), which indicates that scES may provide a benefit to voiding after SCI, which is consistent with previous data showing that scES in humans had benefits for bladder function even if not targeted to the urinary-specific circuits of the cord^25–30^.

The lack of EUS bursting activity in the male and female transected animals is consistent with previous studies that utilized the standard surgical dose (1.2 g/kg) of urethane^42–44^, a dosage necessary for the current acute terminal mapping studies. It has been shown that when animals are tested without anesthesia, some SCI animals do have EUS bursting activity^45,46^. Also, immediately after spinal transection, a reduction in the amount of anesthesia needed has been shown when compared to uninjured animals^47^. Furthermore, one past study that used 1.2 g/kg urethane for intact control animals and an incomplete dose of 0.8 g/kg for animals with SCI was able to detect bursting activity after chronic spinal transection^48^. Therefore, when chronic spinal transection is paired with a dosage that maintains a surgical plane of anesthesia (1.2 g/kg urethane) as in the current experiments, depressive effects on some reflex activity such as bursting of the EUS can occur.

Responses in male rats, both intact and transected, showed atypical activity and responses even at baseline whereby there were numerous rapid voids of small volume (up to 4 separate voids within 120 s). This hyperactivity is most likely related to a necessary seminal vesicle ligation that was performed to prevent occlusion by seminal plug material. Other experiments in our lab (published^33^ and unpublished data) had multiple males with partially or completely occluded urethral outlets, especially after transection and scES at lumbar levels. To prevent loss of data due to urethral occlusion from plug material, the seminal vesicles were ligated, and the urethra flushed, a process which eliminated urethral blockages during experimentation. However, it is possible (if not likely) that some branches of the hypogastric nerve^49^ were impacted by this process, leading to weakened efficacy of scES relative to females where such manipulations were not done.

### scES-induced colorectal effects

T13-L2 scES, at 6 weeks post-injury, produced inhibition of rectal contractions, a finding consistent with scES of the L5-S1 spinal cord^33^. Furthermore, this result is consistent with previous literature^50,51^ on the role of the autonomic (hypogastric) nerves in rectal motility. Importantly, L5-S1 scES should influence the activity of the lumbosacral trunk (and so the pelvic nerve) which should increase activity in the rectum^50,51^. Therefore the observation of inhibition, in this and the previous study, suggests scES is activating an integrated spinal circuit whose ultimate functional output cannot be predicted by sympathetic/parasympathetic characterizations.

Distal colon activity was found to increase (contraction frequency) due to T13-L2 scES. As above, distal colon activity has been found previously to^50,51^ be reduced from sympathetic stimulation and therefore this result would not be predicted by a simple sympathetic characterization. Although, it has been shown that sympathetic stimulation has differential effects on distal colon function according to the muscle layer (inhibited longitudinal activity but increased circular activity)^52^. Therefore, this result could be due to the way in which activity was measured, a pressure probe within the lumen, and therefore more likely to detect circular muscle effects rather than longitudinal muscle effects of T13-L2 stimulation.

## Limitations and future directions

The large surface area of the modified 5-6-5 Medtronic electrode, in comparison to the rat spinal cord, limits the spatial specificity of the electrical stimulation. It may be that an electrode array with multiple contacts in the same surface area would enable better separation of bladder and bowel responses and reduce or eliminate accompanying movements, as demonstrated with a Micro-Leads 15-electrode array^33^, which is currently being used in our ongoing studies. Obstruction of the urethra in male rats due to the presence of seminal plug material is a limitation of scES in male rats. Although the blockage was resolved with seminal vesicle ligation and flushing of the urethra prior to testing, the procedure likely was responsible for weakened responses (see above) and should be avoided in future studies. Stimulus presentation was only partially randomized and therefore may have led to carry over effects that could not be distinguished from bladder distension, which is unavoidable during standard cystometrogram testing. As discussed above, the urethane dosage may have interfered with EUS bursting activity. This dose was chosen to ensure adequate anesthesia depth through a long (2–3 h) and invasive surgical preparation as well as a long testing period (> 3 h) that involved stimulation of the spinal cord at multiple intensities. Such effects will be alleviated in future studies with chronic implants and awake testing experiments.

Future studies should: utilize an electrode array with multiple contacts to better determine site specificity of these effects as well as determine sub-motor threshold effects; determine the response of the bladder and bowel based on the state in which stimulation is applied (full vs. empty), as this could have important consequences for the translation of stimulation technologies aimed at regulating these organs in humans; randomly vary stimulation parameters to confirm these effects and distinguish any carry over effects; and investigate whether scES has a relationship between increased capacity and reduced efficiency as seen by other groups^53^. Additionally, future experiments should be designed to examine additional translationally relevant variables: feasibility/efficacy of chronic electrode implantation; the effect of stimulation on complication progression when applied at various times in recovery (acute, subacute, chronic); what effect repeated stimulation has on bladder and bowel outcome measures; and possible long-term stimulation effects on surrounding tissue.

## Conclusions

Thoracolumbar scES was found to produce a hold effect (inhibition of bladder contractions, reduced volume without OI) in intact animals regardless of sex, whereas the same stimulation produced a void effect (short-latency bladder contraction, reduced EUS activity) in transected animals. This combination of effects in transected animals would benefit individuals with high levels of DSD as it stimulates both bladder contractions and relaxation of the EUS.

## Methods

### Animals

All procedures were approved by the Institutional Use and Care Committee at the University of Louisville School of Medicine and conformed to NIH guidelines and is reported according to ARRIVE guidelines. Animals were assigned to groups upon arrival to the facility without randomization or blinding of experimenter. Groups were: intact female (n = 11, final n = 7), intact male (n = 12, final n = 12), transected female (n = 9, final n = 8), and transected male (n = 12, final n = 9), totaling 24 male and 20 female rats. Reasons for exclusion were: IF—no fluid expelled for 30 min n = 1, possible spinal cord compression n = 1, missing data n = 1, anesthetic death n = 1; STxF—died prior to testing n = 1; and STxM—died during surgery n = 1, no void for 35 min n = 1, equipment malfunction n = 1. Chronically transected Wistar rats (90–150 days old, 6 weeks post-injury) received a complete T9 spinal cord transection; intact animals had no surgical experience. Our previous study found significance with at least n = 8, therefore, the target sample size was chosen to replicate and extend previous findings. Because of the physiological nature of the mapping procedures (without behavioral expression being measured) and the anesthetized preparation, we did not consider treatment application or cage location possible confounds and therefore they were not controlled.

### SCI

Spinal cord injury was previously described^33^. After being anesthetized with ketamine/xylazine (80/10 mg/kg, IP) animals underwent a laminectomy (T8) and cord transection with microdissection scissors. Transection was confirmed visually and the space packed with Gelfoam. Wound closure used 4-0 Ethicon suture in the muscle layer and cutaneous wound clips (Mikrotek, 9 mm autoclip). Post-SCI care: daily bladder emptying (3/day for 7 days, 2/day until voiding reflexively); pain reduction (Meloxicam,1/day for 3 days); and antibiotics (Gentafuse, gentamiacin, SC, 1/day for 3 days; Penject, penicilin G, SC, 1/day for 3 days).

### Anesthesia

At 6 weeks post-injury, animals were anesthetized using Isoflurane (3%). A heated water pad (Gaymar) was used throughout all procedures. Via jugular catheter, the animal was weaned from isoflurane to urethane anesthesia (1.2 g/kg, IV) over approximately 10 min, as described previously^33^. Anesthetic depth was judged by: respiration rate, corneal reflex response. Supplements (urethane, 25 mg IV) given as necessary. All animal groups received the same dose as intact control animals (1.2 g/kg) due to the invasiveness of the procedures and the duration of testing.

### Jugular catheter

In a supine position, a midline incision was used for the placement of both jugular and tracheal catheters. Blunt dissection allowed access to structures. Micro scissors were used to incise the jugular vein and a catheter (PE-10) was inserted. Suture tied the vessel wall securely to the catheter.

### Tracheal catheter

Once exposed, the trachea was transversely cut to open the airway. A tracheal catheter (Y-shaped tubing, with a short extension tubing) was inserted and secured with suture (silk, 4-0). The skin was closed with cyanoacrylate glue.

### Abdominal preparation

A ventral midline cutaneous incision exposed the abdominal muscles. Blunt dissection separated the abdominal muscles to access the organs. This incision was used to: ligate seminal vesicles in males, implant EUS electrodes, and catheterize the dome of the bladder.

### Seminal vesicle ligation

Seminal fluid contains seminal vesicle material that hardens when exposed to prostatic enzymes^54–57^. To prevent occlusion of the urethra, the seminal vesicles were ligated (4-0 silk suture).

### EUS electrodes

A custom surgical table (polycarbonate table with pegs to secure nylon strings with blunted copper hooks) allowed traction to expose the EUS. Fine wire electrodes (0.002 stainless steel, teflon coated, AM Systems), spooled to produce a circular strain reducer, were sutured (6-0 ethylon) to the inguinal ligament, leaving 1–1.5 cm of loose wire for insertion. The end of the wire was cut at a severe angle and ~ 2–3 mm of insulation was stripped for bilateral insertion into the EUS.

### Bladder catheter

Bladder catheterization methods are described elsewhere^33^. The bladder dome was incised using an 18-gauge needle and a catheter (length of PE-60 tubing, flame flared end) was inserted and secured with suture (4-0). Wound closure was done using continuous sutures.

### Laminectomy and implant

A prone position allowed access to the vertebrae. The size of the electrode (~ 3 mm W × 15 mm L) required a quadruple laminectomy for two electrodes (each contact was 2 mm W × 4 mm L) to contact the epidural surface (one anode and one stimulating cathode) along the midline. The laminectomy procedure is described elsewhere^33^. Briefly, sharp and blunt dissection were used to access the vertebrae and fine-tip rongeurs removed the lamina. The muscle layer was closed, after placement and visual confirmation of electrode contact, using suture (4-0) and the cutaneous layer closed with wound clips. The cathode was placed on the area of interest (T13-L2).

### Anorectal manometry (ARM) probes

As described previously^33^, ARM probes were placed at 2 and 10 cm from the anal verge. A lubricated (Surgilube) length of firm tubing (P-80) served as a speculum. The ARM sensor (Millar, 3.5F, SPR-524) was placed at 10 cm and taped to the tail. Finally, the 2 cm sensor was inserted and secured. Millar probes were developed to measure blood pressure but have been shown to work well for measuring colonic contractions in rats^33,58^. ARM probe sensors utilize a plastic membrane that transduces changes in pressure when impinged upon.

### Mapping study

Mapping was conducted according to previously published protocols^33^. After connection to all equipment, the pump infused saline via bladder catheter (0.25 ml/min). Initial acclamation allowed for the bladder to show consistent baseline activity. Once consistent activity (roughly equal ICI) occurred, 5 baseline periods were collected. The ICI determined the testing paradigm: if the ICI was short (~ 30 s–1 min) a period length of 2 min was used; if the ICI was longer than 2 min, a void-to-void paradigm was used. Animals with overflow incontinence had 2 min periods.

Parameter combinations varied intensity (50, 75, 100, 150, and 300 µA) and frequency (5, 10, 30, 45, and 60 Hz). In half of the animals the intensity was varied first (i.e. 5 Hz 50 µA, 5 Hz 75 µA) until all combinations were tested. The other subjects had the frequency varied first (I.e. 5 Hz 50 µA, 10 Hz 50 µA). Each stimulation period (Stim On) was followed by a no stimulation period (Stim Off). Other stimulation parameters: 1 ms pulse duration; no delay; 1 train/second; and 500 ms train duration (500 ms on/500 ms off). Extra Stim Off periods were conducted as neccessary until an approximate return to baseline occurred.

### Acquisition equipment

Data acquisition used a Micro1401 (CED) unit and Spike 2 software (version 8.15, CED). ARM pressures used a control unit (Millar) that fed into the Micro1401. Bladder pressure was recorded with a pressure transducer (World Precision Instruments [WPI, LLC]; Sarasota, FL, USA), void volume with a weight transducer (WPI, LLC), and signals were amplified (WPI, Transbridge 4M, TBM4M, 4 channel pressure amplifier) prior to acquisition. EMG signals were amplified (AM Systems, 4 channel differential amplifier) prior to acquisition.

### Perfusion and histology

Following mapping, scES was used to determine motor thresholds for visualized movements. After thresholding, instrumentation was removed. Electrode pad locations were marked with suture knots at the caudal and rostral aspects of each contact. Anesthetic overdose (1.2 g/kg, 50%, IV) was followed by transcardial perfusion (300 ml, 0.9% heparanized phosphate buffered saline) and fixation (300 ml, 4% phosphate buffered paraformaldehyde). Fixed tissue was collected, and the electrode placement confirmed using the spinal nerves, the next day. Tissue was post-fixed for 1 day, then put in 30% sucrose for sectioning. Sectioning used a cryostat and was thaw-mounted on slides to confirm complete transection. Tissue was stained with Luxol fast blue and crestyl violet per published protocols.

### Quantification

A custom Matlab program quantified data traces (Fig. 1c,d). Quantification: CMG—void volume (ml), bladder pressure (mmHg), maximum contractile pressure (mmHg), ICI (seconds), AUC of contraction (mmHg/second), and contraction time (seconds); EUS EMG—total duration (seconds), tonic activity duration (seconds), bursting duration (milliseconds), and maximum amplitude (mV); and ARM—contraction count within bouts, contraction count non-bout, average amplitude (mmHg), maximum amplitude (mmHg), and contraction frequency (Hz). Other observations (VisMvt) were recorded manually.

### EMG processing

A continuous wavelet transform approach was used to extract EMG signals that correspond to the 60–500 Hz frequency range. Persistent stimulation artifact, especially at higher intensities, were removed by using spikes-removal-interpolation approach, using stim pulse markers.

### Definitions

Bladder contraction: a steep rise in CMG pressure associated with release of fluid from the urethra. Inflection points, abrupt slope changes at the onset and offset of contractions, were used as the start and end points of contractions. These points allowed quantification of outcome measures. Acontractile traces used onset and offset of stimulation. Bladder outcomes included: VV—voided volume was the amount of fluid expelled from the urethra during that period; ICI—the intercontraction interval is the time between the end of one contraction and the end of the next contraction yielding the total time of a fill-void cycle; AUC—area under the curve is a way to compare the amount of activity over the entire testing period by calculating the amount of area between the resultant curve and the zero point of the graph; CT--- contraction time is the duration of a void contraction; and contraction pressure results—these compare the pressures (mean, highest, or lowest) found in that period. EMG used a calculated baseline amplitude and a threshold at twice the background level. EMG outcomes: activity time—is the total time that the signal is above the threshold and represents both tonic and bursting activity; bursting duration—is the duration of each fast burst of activity, bursting frequency—is the frequency of bursts during phasic activity in hertz, ratio of bursting to tonic activity—is a comparison between how much of the total activity is bursting or tonic activity. Bowel contractions were spikes of twice the baseline pressure. Bouts of contractions were groups of contractions with less than 2 s between contractions. Bowel outcomes: contraction amplitude—all contraction amplitude measures (mean, max, min) are the pressure that was recorded during that period; duration—is the summated amount of time during which the tissue was contracting during a testing period; range of pressure—is the difference between the highest and lowest values in that period as a measure of contractile ability of the tissue; AUC—similarly to the area under the curve for bladder function, this is used to compare the overall activity in a period by summing the area of all of the contractions in that period; frequency—refers to the number of contractions within a period, reported in hertz or contractions per second; contraction within bout—this measure sums the number of contractions that occur in bouts during a period; and non-bout contractions—sums the number of contractions that occur outside of bouts.

### Heat map creation

Heatmaps were created by making a two-dimensional matrix with all possible frequency and intensity pairs. This matrix was filled with the average value for the given outcome measure. A fine 2D mesh was applied to the matrix and a spline interpolation created a fine matrix of smooth transitions. The matrix was color-coded for the mean values.

### Parameter divisions

VisMvt Results shows the movement thresholds (lowest intensity that caused muscle contraction) were found to be below 150 µA. The data collected here is consistent previous findings^33^ and supports the intensity division (Q1,2 vs, Q3,4). The frequency dimension was divided based on: scES in the L5/S1 region^33^ and frequencies of 30 Hz and above facilitating voiding in humans^28^.

### Statistical analysis

Data was analyzed using mixed linear models regressing the outcomes on sex, injury (no = Intact, yes = transected), stimulation (yes/no), stimulation frequency and intensity as well as their interactions. To capture within entity variability, a random intercept was added for each rat. Random slopes for stimulation, stimulation frequency and stimulation intensity were also included to capture individual trends. Outcomes were presented by least square means and standard error. Differences studied were obtained by building linear contrasts on the Sex * Stimulation * Injury * Frequency * Intensity * interaction. Prior to statistical analysis, extreme outliers (± 3 interquartile ranges) were excluded. The significance level was p ≤ 0.05, 2-tailed. Statistical analyses were performed in SAS 9.4 (SAS Inc, Cary, NC).

## Supplementary Information

Supplementary Tables.

## Data Availability

As part of the NIH SPARC Materials Sharing policy, the curated datasets generated and/or analyzed for the current study are available at https://doi.org/10.26275/er7m-gir3.
