# Supplementary material for: Thoracolumbar epidural stimulation effects on bladder and bowel function in uninjured and chronic transected anesthetized rats
Source: Sci Rep. 2022 Feb 8;12:2137. doi: 10.1038/s41598-022-06011-2 (PMC8826941; doi:10.1038/s41598-022-06011-2)
Supplement: Supplementary file 1 — Supplementary Tables. [file 41598_2022_6011_MOESM1_ESM.pdf]

Supplement for:

Thoracolumbar epidural stimulation effects on bladder and bowel function in uninjured and chronic transected anesthetized rats.

Robert F. Hoey, Daniel Medina-Aguiñaga, Fahmi Khalifa, Beatrice Ugiliweneza, Dengzhi Wang, Sharon Zdunowski, Jason Fell, Ahmed Naglah, Ayman S. El-Baz, April N. Herrity, Susan J. Harkema, and Charles H. Hubscher.

Supplemental Table 1 - Bladder Function Outcomes (Contractile bladder)					
	scES ON			Between group statistically significant results:	
	Intact Female (n = 7)	Intact Male (n = 12)	Transected Female (n = 5)	IF vs. STxF	IF vs. IM
CT (seconds)	OA: 21.18 (1.3)<br>Q1: 24.39 (1.68)<br>Q2: 22.33 (1.54)<br>Q3: 13.12 (2.32)<br>Q4: 14.82 (2.58)	OA: 19.39 (1.17)<br>Q1: 19.62 (1.44)<br>Q2: 17.92 (1.33)<br>Q3: 24.15 (1.5)<br>Q4: 18.64 (1.6)	OA: 21.41 (1.38)<br>Q1: 23.23 (1.89)<br>Q2: 21.75 (1.75)<br>Q3: 18.46 (1.98)<br>Q4: 19.31 (2.15)	Q3<br>STxF > IF	Q1-3<br>IF > IM
Max CMG pressure (mmHg)	OA: 24.89 (1.73)<br>Q1: 25.6 (1.78)<br>Q2: 24.6 (1.68)<br>Q3: 34.29 (2.23)<br>Q4: 21.96 (2.43)	OA: 30.11 (1.65)<br>Q1: 30.31 (1.62)<br>Q2: 29.14 (1.55)<br>Q3: 29.84 (1.66)<br>Q4: 27.72 (1.73)	OA: 30.91 (1.78)<br>Q1: 32.61 (1.92)<br>Q2: 31.35 (1.82)<br>Q3: 27.63 (1.99)<br>Q4: 26.18 (2.11)	OA, Q1, Q2<br>STxF > IF<br>Q3<br>IF > STxF	OA, Q1-4<br>IM > IF
AUC (mmHg*sec)	OA: 349.29 (30.15)<br>Q1: 355.71 (37.26)<br>Q2: 305.13 (34.15)<br>Q3: 514.07 (53.6)<br>Q4: 280.45 (59.17)	OA: 377.75 (26.99)<br>Q1: 362.78 (31.72)<br>Q2: 319.79 (29.27)<br>Q3: 493.86 (33.6)<br>Q4: 348.36 (35.78)	OA: 447.13 (32.14)<br>Q1: 483.68 (42.11)<br>Q2: 418.57 (39.19)<br>Q3: 356.9 (44.67)<br>Q4: 384.38 (48.18)	OA, Q1, Q2<br>STxF > IF<br>Q3<br>IF > STxF	N.S.
	scES OFF				
CT (seconds)	OA: 21.86 (1.3)<br>Q1: 21.56 (1.68)<br>Q2: 22.89 (1.54)<br>Q3: 18.74 (2.32)<br>Q4: 19.74 (2.58)	OA: 18.77 (1.17)<br>Q1: 20.12 (1.44)<br>Q2: 17.8 (1.33)<br>Q3: 17.66 (1.5)<br>Q4: 19.51 (1.6)	OA: 21.89 (1.38)<br>Q1: 20.47 (1.89)<br>Q2: 21.34 (1.75)<br>Q3: 24.2 (1.98)<br>Q4: 19.91 (2.15)	Q3<br>STxF > IF	OA, Q2<br>IF > IM
Max CMG pressure (mmHg)	OA: 27.01 (1.73)<br>Q1: 25.53 (1.78)<br>Q2: 23.9 (1.68)<br>Q3: 42.74 (2.23)<br>Q4: 39.07 (2.43)	OA: 30.16 (1.65)<br>Q1: 30.35 (1.62)<br>Q2: 28.93 (1.55)<br>Q3: 28.69 (1.66)<br>Q4: 28.91 (1.73)	OA: 31.02 (1.78)<br>Q1: 30.01 (1.92)<br>Q2: 30.01 (1.82)<br>Q3: 31.97 (1.99)<br>Q4: 28.38 (2.11)	OA, Q1, Q2<br>STxF > IF<br>Q3, Q4<br>IF > STxF	OA, Q1-4<br>IM > IF
AUC (mmHg*sec)	OA: 345.92 (30.15)<br>Q1: 303.1 (37.26)<br>Q2: 308.27 (34.15)<br>Q3: 531.35 (53.6)<br>Q4: 481.93 (59.17)	OA: 351.92 (26.99)<br>Q1: 375.18 (31.72)<br>Q2: 314.79 (29.27)<br>Q3: 293.38 (33.6)<br>Q4: 333.07 (35.78)	OA: 445.43 (32.14)<br>Q1: 418.73 (42.11)<br>Q2: 400.74 (39.19)<br>Q3: 447.94 (44.67)<br>Q4: 393.72 (48.18)	OA, Q1, Q2<br>STxF > IF	Q1<br>IM > IF<br>Q3, Q4<br>IF > IM

Supplemental Table 1 - Bladder Function Outcomes (Non-contractile bladder)			
	**scES ON**		**Between group statistically significant results:**
	**Transected Female (n = 3)**	**Transected Male (n = 9)**	**STxF vs. STxM**
AUC (mmHg*sec)	OA: 2225.44 (280.66)<br>Q1: 2803.44 (330.47)<br>Q2: 2794.18 (312.78)<br>Q3: 1514.82 (344.12)<br>Q4: 2016.57 (367.28)	OA: 3062.26 (228.96)<br>Q1: 3370.89 (245.18)<br>Q2: 3414.83 (234.52)<br>Q3: 2684.95 (288.41)<br>Q4: 2785.44 (307.9)	OA, Q2, Q3<br>STxM > STxF
Mean pressure (mmHg)	OA: 22.35 (1.56)<br>Q1: 22.13 (1.62)<br>Q2: 22.65 (1.6)<br>Q3: 20.02 (1.67)<br>Q4: 22.18 (1.7)	OA: 28.81 (1.52)<br>Q1: 28.56 (1.55)<br>Q2: 28.81 (1.54)<br>Q3: 28.4 (1.72)<br>Q4: 29.64 (1.73)	OA, Q1-4<br>STxM > STxF
Max pressure (mmHg)	OA: 35.89 (1.69)<br>Q1: 32.51 (1.78)<br>Q2: 32.08 (1.75)<br>Q3: 40.75 (1.82)<br>Q4: 34.21 (1.86)	OA: 37.99 (1.63)<br>Q1: 35.64 (1.66)<br>Q2: 35.63 (1.65)<br>Q3: 42.32 (1.79)<br>Q4: 37.41 (1.81)	OA, Q1, Q2, Q4<br>STxM > STxF
Min pressure (mmHg)	OA: 14.86 (2.06)<br>Q1: 15.47 (2.12)<br>Q2: 16.12 (2.1)<br>Q3: 11.25 (2.16)<br>Q4: 14.84 (2.19)	OA: 21.72 (2.04)<br>Q1: 22.15 (2.06)<br>Q2: 22.95 (2.05)<br>Q3: 19.84 (2.21)<br>Q4: 22.27 (2.22)	OA, Q1-4<br>STxM > STxF
	**scES OFF**		
AUC (mmHg*sec)	OA: 3479.17 (280.66)<br>Q1: 2811.7 (330.47)<br>Q2: 2920.3 (312.78)<br>Q3: 4442.58 (344.12)<br>Q4: 3312.89 (367.28)	OA: 3915.27 (228.96)<br>Q1: 3341.63 (245.18)<br>Q2: 3565.59 (234.52)<br>Q3: 4532.09 (288.41)<br>Q4: 4077.92 (307.9)	OA, Q2<br>STxM > STxF
Mean pressure (mmHg)	OA: 21.75 (1.56)<br>Q1: 21.25 (1.62)<br>Q2: 22.12 (1.6)<br>Q3: 20.27 (1.67)<br>Q4: 22.01 (1.7)	OA: 27.69 (1.52)<br>Q1: 28.17 (1.55)<br>Q2: 28.7 (1.54)<br>Q3: 26.77 (1.72)<br>Q4: 27.19 (1.73)	OA, Q1-4<br>STxM > STxF
Max pressure (mmHg)	OA: 32.14 (1.69)<br>Q1: 31.37 (1.78)<br>Q2: 31.88 (1.75)<br>Q3: 31.32 (1.82)<br>Q4: 30.76 (1.86)	OA: 35.4 (1.63)<br>Q1: 35.23 (1.66)<br>Q2: 35.24 (1.65)<br>Q3: 35.78 (1.79)<br>Q4: 35.19 (1.81)	OA, Q1-4<br>STxM > STxF
Min pressure (mmHg)	OA: 14.96 (2.06)<br>Q1: 15.73 (2.12)<br>Q2: 16.2 (2.1)<br>Q3: 12.03 (2.16)<br>Q4: 14.35 (2.19)	OA: 21.39 (2.04)<br>Q1: 22.02 (2.06)<br>Q2: 22.72 (2.05)<br>Q3: 20.08 (2.21)<br>Q4: 20.77 (2.22)	OA, Q1-4<br>STxM > STxF

Supplemental Table 1 - Bladder Function Outcomes (EUS EMG)					
	**scES ON**			**Between group statistically significant results:**	
	**Intact Female (n = 7)**	**Intact Male (n = 12)**	**Transected Female (n = 8)**	**IF vs. STxF**	**IF vs. IM**
Mean activity time (seconds)	OA: 11.59 (0.87)<br>Q1: 11.45 (1.05)<br>Q2: 13.76 (0.97)<br>Q3: 6.82 (1.37)<br>Q4: 9.17 (1.51)	OA: 6.79 (0.81)<br>Q1: 7.28 (0.92)<br>Q2: 7.64 (0.87)<br>Q3: 6.04 (0.95)<br>Q4: 5.95 (1.00)	OA: 12.07 (0.91)<br>Q1: 11.99 (1.15)<br>Q2: 13.29 (1.08)<br>Q3: 12.21 (1.2)<br>Q4: 11.69 (1.28)	N.S.	OA, Q1, Q2, Q4<br>IF > IM
Mean burst time (msecs)	OA: 74.49 (1.82)<br>Q1: 72.61 (1.95)<br>Q2: 73.61 (1.87)<br>Q3: 73.93 (2.6)<br>Q4: 77.59 (2.51)	OA: 76.05 (1.74)<br>Q1: 76.44 (1.82)<br>Q2: 75.91 (1.76)<br>Q3: 77.61 (2.14)<br>Q4: 74.17 (2.01)	N.A.	N.A.	Q1, Q2<br>IM > IF
Mean burst frequency (Hz)	OA: 3.9 (0.34)<br>Q1: 6.01 (0.36)<br>Q2: 5.57 (0.34)<br>Q3: 1.63 (0.45)<br>Q4: 3.13 (0.49)	OA: 4.65 (0.3)<br>Q1: 5.82 (0.33)<br>Q2: 5.75 (0.32)<br>Q3: 3.19 (0.34)<br>Q4: 4.24 (0.35)	N.A.	N.A.	OA, Q3, Q4<br>IM > IF
Burst to Tonic ratio	OA: 0.65 (0.06)<br>Q1: 0.8 (0.06)<br>Q2: 0.73 (0.06)<br>Q3: 0.56 (0.1)<br>Q4: 0.55 (0.08)	OA: 0.72 (0.05)<br>Q1: 0.86 (0.05)<br>Q2: 0.82 (0.05)<br>Q3: 0.55 (0.08)<br>Q4: 0.71 (0.07)	N.A.	N.A.	N.S.
	**scES OFF**				
Mean activity time (seconds)	OA: 12.4 (0.87)<br>Q1: 11.94 (1.04)<br>Q2: 13.67 (0.97)<br>Q3: 11.52 (1.37)<br>Q4: 12.9 (1.51)	OA: 7.57 (0.81)<br>Q1: 7.58 (0.92)<br>Q2: 7.55 (0.87)<br>Q3: 7.82 (0.95)<br>Q4: 8.17 (1.01)	OA: 11.42 (0.91)<br>Q1: 11.19 (1.15)<br>Q2: 11.75 (1.08)<br>Q3: 13.24 (1.2)<br>Q4: 11.25 (1.29)	N.S.	OA, Q1-4<br>IF > IM
Mean burst time (msecs)	OA: 74.68 (1.82)<br>Q1: 74.06 (1.95)<br>Q2: 72.86 (1.87)<br>Q3: 72.51 (2.27)<br>Q4: 79.25 (2.44)	OA: 75.66 (1.7)<br>Q1: 75.33 (1.82)<br>Q2: 75.52 (1.76)<br>Q3: 76.28 (1.84)<br>Q4: 76.22 (1.9)	N.A.	N.A.	Q2, Q3<br>IM > IF
Mean burst frequency (Hz)	OA: 5.42 (0.34)<br>Q1: 6.23 (0.36)<br>Q2: 5.74 (0.34)<br>Q3: 4.55 (0.45)<br>Q4: 5.2 (0.49)	OA: 5.67 (0.3)<br>Q1: 5.79 (0.33)<br>Q2: 5.78 (0.32)<br>Q3: 5.69 (0.34)<br>Q4: 5.43 (0.35)	N.A.	N.A.	Q3<br>IM > IF
Burst to Tonic ratio	OA: 0.72 (0.06)<br>Q1: 0.86 (0.06)<br>Q2: 0.76 (0.06)<br>Q3: 0.54 (0.09)<br>Q4: 0.69 (0.09)	OA: 0.8 (0.05)<br>Q1: 0.82 (0.05)<br>Q2: 0.8 (0.05)<br>Q3: 0.77 (0.06)<br>Q4: 0.76 (0.06)	N.A.	N.A.	Q3<br>IM > IF

Supplemental Table 2 - Rectum Anorectal Manometry Outcomes

	**scES ON**				**Between group statistically significant results:**			
	**Intact Female (n = 7)**	**Intact Male (n = 12)**	**Transected Female (n = 8)**	**Transected Male (n = 9)**	**IF vs. STxF**	**IF vs. IM**	**IM vs. STxM**	**STxF vs. STxM**
Mean amplitude (mmHg)	OA: 0.85 (0.07)<br>Q1: 0.72 (0.09)<br>Q2: 0.86 (0.08)<br>Q3: 1.23 (0.12)<br>Q4: 0.68 (0.13)	OA: 0.83 (0.07)<br>Q1: 0.78 (0.08)<br>Q2: 0.92 (0.07)<br>Q3: 0.72 (0.08)<br>Q4: 0.77 (0.08)	OA: 0.5 (0.07)<br>Q1: 0.47 (0.08)<br>Q2: 0.56 (0.08)<br>Q3: 0.6 (0.09)<br>Q4: 0.32 (0.09)	OA: 0.44 (0.07)<br>Q1: 0.32 (0.08)<br>Q2: 0.41 (0.08)<br>Q3: 0.62 (0.1)<br>Q4: 0.43 (0.11)	OA, Q1-3<br>IF > STxF	Q3<br>IF > IM	OA, Q1,Q2,Q4<br>IM > STxM	Q2<br>STxF > STxM
Max amplitude (mmHg)	OA: 1.13 (0.14)<br>Q1: 0.93 (0.17)<br>Q2: 1.19 (0.16)<br>Q3: 1.7 (0.23)<br>Q4: 0.98 (0.25)	OA: 1.46 (0.12)<br>Q1: 1.38 (0.14)<br>Q2: 1.68 (0.13)<br>Q3: 1.24 (0.15)<br>Q4: 1.14 (0.16)	OA: 0.84 (0.13)<br>Q1: 0.72 (0.16)<br>Q2: 1.07 (0.15)<br>Q3: 0.84 (0.17)<br>Q4: 0.6 (0.18)	OA: 0.71 (0.13)<br>Q1: 0.49 (0.16)<br>Q2: 0.7 (0.14)<br>Q3: 1.01 (0.2)<br>Q4: 0.64 (0.22)	OA, Q3<br>IF > STxF	OA, Q1,Q2<br>IM > IF	OA, Q1,Q2,Q4<br>IM > STxM	Q2<br>STxF > STxM
Mean AUC (mmHg s)	OA: 2.4 (0.33)<br>Q1: 1.98 (0.38)<br>Q2: 2.62 (0.36)<br>Q3: 2.33 (0.48)<br>Q4: 2.04 (0.51)	OA: 2.5 (0.31)<br>Q1: 2.35 (0.34)<br>Q2: 2.72 (0.32)<br>Q3: 2.11 (0.35)<br>Q4: 2.27 (0.36)	OA: 1.79 (0.32)<br>Q1: 2.18 (0.37)<br>Q2: 2.1 (0.36)<br>Q3: 1.26 (0.38)<br>Q4: 1.35 (0.39)	OA: 2.12 (0.32)<br>Q1: 1.9 (0.36)<br>Q2: 2.17 (0.34)<br>Q3: 2.1 (0.43)<br>Q4: 2.71 (0.46)	OA, Q3<br>IF > STxF	N.S.	OA, Q2<br>IM > STxM	Q3,Q4<br>STxM > STxF
Mean duration (sec)	OA: 3.59 (0.31)<br>Q1: 3.5 (0.39)<br>Q2: 4.07 (0.36)<br>Q3: 2.06 (0.54)<br>Q4: 3.14 (0.59)	OA: 4.18 (0.27)<br>Q1: 4.32 (0.33)<br>Q2: 4.45 (0.31)<br>Q3: 3.5 (0.35)<br>Q4: 4.06 (0.37)	OA: 5.32 (0.29)<br>Q1: 6.67 (0.37)<br>Q2: 6.21 (0.35)<br>Q3: 3.3 (0.39)<br>Q4: 4.29 (0.41)	OA: 3.91 (0.29)<br>Q1: 4.39 (0.36)<br>Q2: 4.2 (0.33)<br>Q3: 2.7 (0.47)<br>Q4: 3.64 (0.51)	OA, Q1-4<br>STxF > IF	OA, Q1,Q3<br>IM > IF	N.S.	Oa, Q1,Q2<br>STxF > STxM
	**scES OFF**							
Mean amplitude (mmHg)	OA: 0.67 (0.07)<br>Q1: 0.56 (0.09)<br>Q2: 0.7 (0.09)<br>Q3: 0.57 (0.12)<br>Q4: 0.54 (0.13)	OA: 0.88 (0.07)<br>Q1: 0.83 (0.08)<br>Q2: 0.94 (0.07)<br>Q3: 0.85 (0.08)<br>Q4: 0.78 (0.08)	OA: 0.51 (0.07)<br>Q1: 0.51 (0.08)<br>Q2: 0.53 (0.08)<br>Q3: 0.49 (0.09)<br>Q4: 0.45 (0.09)	OA: 0.43 (0.07)<br>Q1: 0.34 (0.08)<br>Q2: 0.43 (0.08)<br>Q3: 0.52 (0.1)<br>Q4: 0.37 (0.11)	OA, Q2<br>IF > STxF	OA, Q1-3<br>IM > IF	OA, Q1-4<br>IM > STxM	Q1<br>STxF > STxM
Max amplitude (mmHg)	OA: 0.86 (0.14)<br>Q1: 0.63 (0.17)<br>Q2: 0.92 (0.16)<br>Q3: 0.7 (0.24)<br>Q4: 0.82 (0.25)	OA: 1.62 (0.12)<br>Q1: 1.41 (0.14)<br>Q2: 1.74 (0.13)<br>Q3: 1.6 (0.15)<br>Q4: 1.54 (0.16)	OA: 1.01 (0.13)<br>Q1: 0.99 (0.16)<br>Q2: 1.1 (0.15)<br>Q3: 1.04 (0.17)<br>Q4: 0.79 (0.18)	OA: 0.82 (0.13)<br>Q1: 0.58 (0.16)<br>Q2: 0.83 (0.14)<br>Q3: 0.98 (0.2)<br>Q4: 0.69 (0.22)	N.S.	OA, Q1-4<br>IM > IF	OA, Q1-4<br>IM > STxM	N.S.
Mean AUC (mmHg s)	OA: 2.25 (0.33)<br>Q1: 1.49 (0.38)<br>Q2: 2.33 (0.36)<br>Q3: 2.86 (0.5)<br>Q4: 2.04 (0.52)	OA: 2.65 (0.31)<br>Q1: 2.4 (0.34)<br>Q2: 2.69 (0.32)<br>Q3: 2.79 (0.35)<br>Q4: 2.47 (0.36)	OA: 1.98 (0.32)<br>Q1: 2.18 (0.37)<br>Q2: 1.81 (0.36)<br>Q3: 2.09 (0.38)<br>Q4: 1.86 (0.39)	OA: 2.2 (0.32)<br>Q1: 2.09 (0.36)<br>Q2: 2.1 (0.34)<br>Q3: 2.61 (0.43)<br>Q4: 2.09 (0.46)	Q1<br>STxF > IF	OA, Q1<br>IM > IF	OA, Q2<br>IM > STxM	N.S.
Mean duration (sec)	OA: 3.8 (0.31)<br>Q1: 3.54 (0.39)<br>Q2: 3.82 (0.36)<br>Q3: 3.71 (0.54)<br>Q4: 3.15 (0.59)	OA: 4.37 (0.27)<br>Q1: 4.4 (0.33)<br>Q2: 4.46 (0.31)<br>Q3: 4.35 (0.35)<br>Q4: 4.35 (0.37)	OA: 6.05 (0.29)<br>Q1: 6.27 (0.37)<br>Q2: 5.81 (0.35)<br>Q3: 6.08 (0.39)<br>Q4: 6.24 (0.41)	OA: 4.04 (0.29)<br>Q1: 4.15 (0.36)<br>Q2: 3.71 (0.33)<br>Q3: 4.3 (0.47)<br>Q4: 4.16 (0.51)	OA, Q1-4<br>STxF > IF	OA, Q1,Q2,Q4<br>IM > IF	Q2<br>IM > STxM	OA, Q1-4<br>STxF > STxM

Supplemental Table 2 - Rectum Anorectal Manometry Outcomes continued								
	**scES ON**				**Between group statistically significant results:**			
	**Intact Female (n = 7)**	**Intact Male (n = 12)**	**Transected Female (n = 8)**	**Transected Male (n = 9)**	**IF vs. STxF**	**IF vs. IM**	**IM vs. STxM**	**STxF vs. STxM**
Mean range	OA: 1.32 (0.14)<br>Q1: 1.08 (0.17)<br>Q2: 1.27 (0.16)<br>Q3: 2.24 (0.22)<br>Q4: 1.19 (0.24)	OA: 1.36 (0.13)<br>Q1: 1.28 (0.15)<br>Q2: 1.49 (0.14)<br>Q3: 1.24 (0.15)<br>Q4: 1.21 (0.16)	OA: 0.98 (0.13)<br>Q1: 0.87 (0.16)<br>Q2: 1.14 (0.15)<br>Q3: 1.26 (0.17)<br>Q4: 0.54 (0.18)	OA: 0.86 (0.13)<br>Q1: 0.65 (0.16)<br>Q2: 0.8 (0.15)<br>Q3: 1.26 (0.2)<br>Q4: 0.88 (0.21)	OA, Q3<br>IF > STxF	Q3<br>IF > IM	OA, Q1,Q2<br>IM > STxM	Q2<br>STxF > STxM
Count bout	OA: 5.63 (1.09)<br>Q1: 6.06 (1.38)<br>Q2: 7.41 (1.28)<br>Q3: 5.06 (1.8)<br>Q4: 3.13 (2.01)	OA: 6.66 (0.97)<br>Q1: 7 (1.18)<br>Q2: 7.94 (1.1)<br>Q3: 5.25 (1.21)<br>Q4: 4.5 (1.29)	OA: 4.02 (1.04)<br>Q1: 4.69 (1.32)<br>Q2: 6.07 (1.24)<br>Q3: 2.34 (1.36)<br>Q4: 1.27 (1.46)	OA: 2.87 (1.04)<br>Q1: 1.89 (1.29)<br>Q2: 4.13 (1.19)<br>Q3: 1.18 (1.61)<br>Q4: 0.55 (1.78)	N.S.	N.S.	OA, Q1-4<br>IM > STxM	Q1<br>STxF > STxM
Count non-bout	OA: 4 (0.34)<br>Q1: 3.54 (0.46)<br>Q2: 3.89 (0.41)<br>Q3: 6.08 (0.62)<br>Q4: 4.85 (0.7)	OA: 3.13 (0.29)<br>Q1: 2.84 (0.38)<br>Q2: 3.08 (0.34)<br>Q3: 3.85 (0.4)<br>Q4: 2.85 (0.42)	OA: 3.23 (0.32)<br>Q1: 3.22 (0.43)<br>Q2: 3.46 (0.4)<br>Q3: 3.73 (0.45)<br>Q4: 2.38 (0.48)	OA: 2.09 (0.32)<br>Q1: 1.35 (0.42)<br>Q2: 2.09 (0.38)<br>Q3: 3.29 (0.54)<br>Q4: 0.81 (0.61)	OA, Q3<br>IF > STxF	OA, Q2-4<br>IF > IM	OA, Q1,Q2,Q4<br>IM > STxM	OA, Q1,Q2,Q4<br>STxF > STxM
Contraction frequency (Hz)	OA: 0.07 (0.01)<br>Q1: 0.06 (0.01)<br>Q2: 0.08 (0.01)<br>Q3: 0.08 (0.01)<br>Q4: 0.07 (0.01)	OA: 0.08 (0.01)<br>Q1: 0.08 (0.01)<br>Q2: 0.1 (0.01)<br>Q3: 0.07 (0.01)<br>Q4: 0.06 (0.01)	OA: 0.06 (0.01)<br>Q1: 0.06 (0.01)<br>Q2: 0.07 (0.01)<br>Q3: 0.06 (0.01)<br>Q4: 0.03 (0.01)	OA: 0.05 (0.01)<br>Q1: 0.03 (0.01)<br>Q2: 0.05 (0.01)<br>Q3: 0.05 (0.01)<br>Q4: 0.02 (0.01)	N.S.	OA, Q1,Q2<br>IM > IF	OA, Q1,Q4<br>IM > STxM<br>Q2<br>STxM > IM	OA, Q1,Q2<br>STxF > STxM
	**scES OFF**							
Mean range	OA: 1 (0.14)<br>Q1: 0.77 (0.17)<br>Q2: 0.99 (0.16)<br>Q3: 1.06 (0.23)<br>Q4: 0.78 (0.24)	OA: 1.45 (0.13)<br>Q1: 1.36 (0.15)<br>Q2: 1.54 (0.14)<br>Q3: 1.4 (0.15)<br>Q4: 1.31 (0.16)	OA: 1.04 (0.13)<br>Q1: 1.09 (0.16)<br>Q2: 1.09 (0.15)<br>Q3: 1.03 (0.17)<br>Q4: 0.85 (0.17)	OA: 0.85 (0.13)<br>Q1: 0.71 (0.16)<br>Q2: 0.86 (0.15)<br>Q3: 1 (0.2)<br>Q4: 0.73 (0.21)	Q1<br>STxF > IF	OA, Q1,Q2,Q4<br>IM > IF	OA, Q1-4<br>IM > STxM	OA, Q1<br>STxF > STxM
Count bout	OA: 6.5 (1.1)<br>Q1: 4.26 (1.38)<br>Q2: 8.61 (1.29)<br>Q3: 5.78 (1.8)<br>Q4: 3.95 (2.01)	OA: 7.45 (0.97)<br>Q1: 5.68 (1.18)<br>Q2: 8.27 (1.1)<br>Q3: 8.13 (1.21)<br>Q4: 7.58 (1.31)	OA: 6.45 (1.04)<br>Q1: 6.88 (1.32)<br>Q2: 6.78 (1.26)<br>Q3: 6.49 (1.4)<br>Q4: 4.83 (1.47)	OA: 6.41 (1.04)<br>Q1: 4.69 (1.29)<br>Q2: 7.91 (1.19)<br>Q3: 5.98 (1.61)<br>Q4: 2.3 (1.78)	N.S.	N.S.	Q4<br>IM > STxM	N.S.
Count non-bout	OA: 4.07 (0.34)<br>Q1: 3.22 (0.45)<br>Q2: 4.48 (0.42)<br>Q3: 3.52 (0.62)<br>Q4: 3.32 (0.7)	OA: 3.37 (0.29)<br>Q1: 2.99 (0.38)<br>Q2: 3.58 (0.34)<br>Q3: 3.39 (0.39)<br>Q4: 3.3 (0.42)	OA: 3.55 (0.32)<br>Q1: 3.34 (0.43)<br>Q2: 3.28 (0.4)<br>Q3: 4.16 (0.45)<br>Q4: 3.26 (0.5)	OA: 1.92 (0.32)<br>Q1: 1.42 (0.42)<br>Q2: 1.88 (0.38)<br>Q3: 2.62 (0.54)<br>Q4: 1.62 (0.61)	Q2<br>IF > STxF	OA, Q2<br>IF > IM	OA, Q1,Q2,Q4<br>IM > STxM	OA, Q1-4<br>STxF > STxM
Contraction frequency (Hz)	OA: 0.06 (0.01)<br>Q1: 0.05 (0.01)<br>Q2: 0.07 (0.01)<br>Q3: 0.05 (0.01)<br>Q4: 0.04 (0.01)	OA: 0.08 (0.01)<br>Q1: 0.08 (0.01)<br>Q2: 0.1 (0.01)<br>Q3: 0.08 (0.01)<br>Q4: 0.07 (0.01)	OA: 0.07 (0.01)<br>Q1: 0.07 (0.01)<br>Q2: 0.08 (0.01)<br>Q3: 0.07 (0.01)<br>Q4: 0.05 (0.01)	OA: 0.06 (0.01)<br>Q1: 0.05 (0.01)<br>Q2: 0.08 (0.01)<br>Q3: 0.05 (0.01)<br>Q4: 0.03 (0.01)	Q1<br>STxF > IF	OA, Q1-4<br>IM > IF	OA, Q1-4<br>IM > STxM	N.S.

Supplemental Table 3 - Distal Colon Anorectal Manometry Outcomes								
	**scES ON**				**Between group statistically significant results:**			
	**Intact Female (n = 7)**	**Intact Male (n = 12)**	**Transected Female (n = 8)**	**Transected Male (n = 9)**	**IF vs. STxF**	**IF vs. IM**	**IM vs. STxM**	**STxF vs. STxM**
Mean amplitude (mmHg)	OA: 1.39 (0.09)<br>Q1: 1.47 (0.12)<br>Q2: 1.39 (0.11)<br>Q3: 1.39 (0.15)<br>Q4: 1.28 (0.17)	OA: 0.76 (0.08)<br>Q1: 0.65 (0.1)<br>Q2: 0.79 (0.09)<br>Q3: 0.89 (0.1)<br>Q4: 0.76 (0.11)	OA: 0.6 (0.09)<br>Q1: 0.54 (0.11)<br>Q2: 0.61 (0.1)<br>Q3: 0.75 (0.12)<br>Q4: 0.5 (0.12)	OA: 0.83 (0.09)<br>Q1: 0.69 (0.11)<br>Q2: 0.77 (0.1)<br>Q3: 1.32 (0.14)<br>Q4: 0.6 (0.15)	OA, Q1-4<br>IF > STxF	OA, Q1-4<br>IF > IM	Q3<br>STxM > IM	OA, Q3<br>STxM > STxF
Max amplitude (mmHg)	OA: 1.93 (0.13)<br>Q1: 1.93 (0.17)<br>Q2: 1.84 (0.16)<br>Q3: 1.96 (0.23)<br>Q4: 2.08 (0.26)	OA: 1.12 (0.11)<br>Q1: 0.9 (0.15)<br>Q2: 1.12 (0.13)<br>Q3: 1.48 (0.15)<br>Q4: 1.04 (0.16)	OA: 1.01 (0.12)<br>Q1: 0.89 (0.16)<br>Q2: 0.92 (0.15)<br>Q3: 1.44 (0.17)<br>Q4: 0.78 (0.19)	OA: 1.28 (0.12)<br>Q1: 1.07 (0.16)<br>Q2: 1.18 (0.15)<br>Q3: 2.47 (0.21)<br>Q4: 0.78 (0.19)	OA, Q1-4<br>IF > STxF	OA, Q1-4<br>IF > IM	Q3<br>STxM > IM	OA, Q3<br>STxM > STxF
Mean AUC (mmHg s)	OA: 4.08 (0.48)<br>Q1: 4.77 (0.65)<br>Q2: 4.69 (0.59)<br>Q3: 2.49 (0.9)<br>Q4: 2.8 (1.01)	OA: 5.49 (0.41)<br>Q1: 5.95 (0.54)<br>Q2: 6.01 (0.49)<br>Q3: 4.22 (0.56)<br>Q4: 4.44 (0.61)	OA: 2.97 (0.45)<br>Q1: 3.26 (0.62)<br>Q2: 4.03 (0.57)<br>Q3: 2.06 (0.65)<br>Q4: 2.02 (0.7)	OA: 4.38 (0.45)<br>Q1: 5.43 (0.6)<br>Q2: 5.47 (0.54)<br>Q3: 3.17 (0.79)<br>Q4: 2.19 (0.88)	OA, Q1<br>IF > STxF	OA, Q2<br>IM > IF	OA, Q4<br>IM > STxM	OA, Q1-2<br>STxM > STxF
Mean duration (sec)	OA: 3.76 (0.29)<br>Q1: 4.15 (0.41)<br>Q2: 4.44 (0.37)<br>Q3: 2.04 (0.58)<br>Q4: 3.33 (0.66)	OA: 5.14 (0.24)<br>Q1: 5.71 (0.33)<br>Q2: 5.63 (0.3)<br>Q3: 3.64 (0.35)<br>Q4: 4.12 (0.38)	OA: 4.34 (0.27)<br>Q1: 5.2 (0.39)<br>Q2: 5.78 (0.35)<br>Q3: 2.59 (0.41)<br>Q4: 3 (0.45)	OA: 4.78 (0.26)<br>Q1: 5.91 (0.37)<br>Q2: 6.52 (0.33)<br>Q3: 2.86 (0.51)<br>Q4: 1.93 (0.57)	OA, Q1-2<br>STxF > IF	OA, Q1-3<br>IM > IF	Q2<br>STxM > IM<br>Q4<br>IM > STxM	N.S.
	**scES OFF**							
Mean amplitude (mmHg)	OA: 1.44 (0.09)<br>Q1: 1.4 (0.12)<br>Q2: 1.44 (0.11)<br>Q3: 1.32 (0.15)<br>Q4: 1.37 (0.17)	OA: 0.71 (0.08)<br>Q1: 0.76 (0.1)<br>Q2: 0.72 (0.09)<br>Q3: 0.66 (0.1)<br>Q4: 0.69 (0.11)	OA: 0.58 (0.09)<br>Q1: 0.58 (0.11)<br>Q2: 0.64 (0.1)<br>Q3: 0.55 (0.11)<br>Q4: 0.48 (0.12)	OA: 0.69 (0.09)<br>Q1: 0.69 (0.11)<br>Q2: 0.78 (0.1)<br>Q3: 0.62 (0.14)<br>Q4: 0.54 (0.15)	OA, Q1-4<br>IF > STxF	OA, Q1-4<br>IF > IM	N.S.	N.S.
Max amplitude (mmHg)	OA: 2.01 (0.13)<br>Q1: 1.84 (0.17)<br>Q2: 2.01 (0.16)<br>Q3: 1.89 (0.23)<br>Q4: 1.97 (0.26)	OA: 1.01 (0.11)<br>Q1: 1.03 (0.15)<br>Q2: 0.98 (0.13)<br>Q3: 0.97 (0.15)<br>Q4: 1 (0.16)	OA: 0.98 (0.12)<br>Q1: 1.08 (0.16)<br>Q2: 1.06 (0.15)<br>Q3: 0.9 (0.17)<br>Q4: 0.73 (0.19)	OA: 1 (0.12)<br>Q1: 1.03 (0.16)<br>Q2: 1.12 (0.15)<br>Q3: 0.96 (0.2)<br>Q4: 0.84 (0.23)	OA, Q1-4<br>IF > STxF	OA, Q1-4<br>IF > IM	N.S.	N.S.
Mean AUC (mmHg s)	OA: 4.37 (0.48)<br>Q1: 4.97 (0.65)<br>Q2: 4.52 (0.59)<br>Q3: 4.19 (0.9)<br>Q4: 3.31 (1.01)	OA: 6.04 (0.41)<br>Q1: 6.38 (0.54)<br>Q2: 5.92 (0.49)<br>Q3: 5.6 (0.56)<br>Q4: 6.82 (0.61)	OA: 3.65 (0.45)<br>Q1: 3.32 (0.62)<br>Q2: 4.1 (0.57)<br>Q3: 4.15 (0.64)<br>Q4: 2.72 (0.7)	OA: 5.11 (0.45)<br>Q1: 5.12 (0.6)<br>Q2: 5.58 (0.54)<br>Q3: 4.74 (0.79)<br>Q4: 3.73 (0.88)	Q1<br>IF > STxF	OA, Q1,Q2,Q4<br>IM > IF	OA, Q4<br>IM > STxM	OA, Q1-2<br>STxM > STxF
Mean duration (sec)	OA: 4.15 (0.29)<br>Q1: 4.46 (0.41)<br>Q2: 4.33 (0.37)<br>Q3: 3.61 (0.58)<br>Q4: 4.19 (0.66)	OA: 5.8 (0.24)<br>Q1: 5.85 (0.33)<br>Q2: 5.87 (0.3)<br>Q3: 5.31 (0.35)<br>Q4: 6.26 (0.38)	OA: 5.27 (0.27)<br>Q1: 5.03 (0.39)<br>Q2: 5.51 (0.35)<br>Q3: 5.65 (0.41)<br>Q4: 5.15 (0.45)	OA: 6.01 (0.26)<br>Q1: 5.58 (0.37)<br>Q2: 6.88 (0.33)<br>Q3: 5.25 (0.51)<br>Q4: 4.18 (0.57)	OA, Q2,Q3<br>STxF > IF	OA, Q1-4<br>IM > IF	Q2<br>STxM > IM<br>Q4<br>IM > STxM	OA, Q2<br>STxM > STxF

Supplemental Table 3 - Distal Colon Anorectal Manometry Outcomes continued								
	**scES ON**				**Between group statistically significant results:**			
	**Intact Female (n = 7)**	**Intact Male (n = 12)**	**Transected Female (n = 8)**	**Transected Male (n = 9)**	**IF vs. STxF**	**IF vs. IM**	**IM vs. STxM**	**STxF vs. STxM**
Mean range	OA: 2.29 (0.15)<br>Q1: 2.36 (0.2)<br>Q2: 2.24 (0.18)<br>Q3: 2.68 (0.26)<br>Q4: 2.08 (0.29)	OA: 1.52 (0.14)<br>Q1: 1.27 (0.17)<br>Q2: 1.55 (0.16)<br>Q3: 1.81 (0.17)<br>Q4: 1.59 (0.19)	OA: 1.12 (0.15)<br>Q1: 1.01 (0.19)<br>Q2: 1.1 (0.18)<br>Q3: 1.46 (0.2)<br>Q4: 0.93 (0.21)	OA: 1.62 (0.15)<br>Q1: 1.44 (0.19)<br>Q2: 1.5 (0.17)<br>Q3: 2.55 (0.23)<br>Q4: 1.16 (0.26)	OA, Q1-3<br>IF > STxF	OA, Q1-3<br>IF > IM	Q3<br>STxM > IM	OA, Q1-3<br>STxM > STxF
Count bout	OA: 4.32 (0.48)<br>Q1: 3.44 (0.74)<br>Q2: 3.94 (0.61)<br>Q3: 3.85 (0.91)<br>Q4: 4.07 (1.01)	OA: 1.31 (0.4)<br>Q1: 0.55 (0.52)<br>Q2: 0.8 (0.48)<br>Q3: 3.07 (0.55)<br>Q4: 2.3 (0.59)	OA: 2.84 (0.43)<br>Q1: 3.41 (0.59)<br>Q2: 2.58 (0.55)<br>Q3: 2.95 (0.62)<br>Q4: 2.47 (0.67)	OA: 2.1 (0.43)<br>Q1: 2.09 (0.58)<br>Q2: 1.93 (0.52)<br>Q3: 3.31 (0.77)<br>Q4: 1.91 (0.84)	OA, Q2<br>IF > STxF	OA, Q1,Q2<br>IF > IM	OA, Q1,Q2<br>STxM > IM	Q2<br>STxF > STxM
Count non-bout	OA: 3.94 (0.45)<br>Q1: 3.79 (0.6)<br>Q2: 3.1 (0.54)<br>Q3: 4.89 (0.83)<br>Q4: 4.75 (0.92)	OA: 3.33 (0.39)<br>Q1: 1.91 (0.5)<br>Q2: 2.75 (0.46)<br>Q3: 6.2 (0.55)<br>Q4: 4.22 (0.56)	OA: 3.78 (0.42)<br>Q1: 3.66 (0.57)<br>Q2: 3.29 (0.53)<br>Q3: 4.22 (0.59)<br>Q4: 4.34 (0.65)	OA: 4.44 (0.42)<br>Q1: 4.35 (0.55)<br>Q2: 4.95 (0.5)<br>Q3: 5.26 (0.72)<br>Q4: 2.42 (0.8)	N.S.	Q1<br>IM > IF	OA, Q1,Q2<br>STxM > IM<br>Q4<br>IM > STxM	Q2<br>STxM > STxF<br>Q4<br>STxF > STxM
Contraction frequency (Hz)	OA: 0.09 (0.01)<br>Q1: 0.1 (0.01)<br>Q2: 0.09 (0.01)<br>Q3: 0.1 (0.01)<br>Q4: 0.08 (0.01)	OA: 0.04 (0.01)<br>Q1: 0.02 (0.01)<br>Q2: 0.04 (0.01)<br>Q3: 0.06 (0.01)<br>Q4: 0.06 (0.01)	OA: 0.07 (0.01)<br>Q1: 0.06 (0.01)<br>Q2: 0.06 (0.01)<br>Q3: 0.08 (0.01)<br>Q4: 0.07 (0.01)	OA: 0.06 (0.01)<br>Q1: 0.06 (0.01)<br>Q2: 0.06 (0.01)<br>Q3: 0.11 (0.01)<br>Q4: 0.05 (0.01)	OA, Q1,Q2<br>IF > STxF	OA, Q1-3<br>IF > IM	OA, Q1-3<br>STxM > IM	Q3<br>STxM > STxF
	**scES OFF**							
Mean range	OA: 2.29 (0.15)<br>Q1: 2.32 (0.2)<br>Q2: 2.28 (0.18)<br>Q3: 2.12 (0.26)<br>Q4: 2.12 (0.29)	OA: 1.45 (0.14)<br>Q1: 1.49 (0.17)<br>Q2: 1.47 (0.16)<br>Q3: 1.37 (0.17)<br>Q4: 1.49 (0.19)	OA: 1.05 (0.15)<br>Q1: 1.06 (0.19)<br>Q2: 1.14 (0.18)<br>Q3: 1.01 (0.19)<br>Q4: 0.87 (0.21)	OA: 1.39 (0.15)<br>Q1: 1.48 (0.19)<br>Q2: 1.53 (0.17)<br>Q3: 1.29 (0.23)<br>Q4: 1.08 (0.26)	OA, Q1-4<br>IF > STxF	OA, Q1-4<br>IF > IM	N.S.	OA, Q1,Q2<br>STxM > STxF
Count bout	OA: 4.33 (0.48)<br>Q1: 4.41 (0.71)<br>Q2: 3.94 (0.63)<br>Q3: 3.57 (0.91)<br>Q4: 4.72 (1.03)	OA: 0.88 (0.4)<br>Q1: 0.78 (0.52)<br>Q2: 0.91 (0.48)<br>Q3: 1.21 (0.55)<br>Q4: 0.8 (0.59)	OA: 2.74 (0.43)<br>Q1: 3.36 (0.59)<br>Q2: 3.37 (0.55)<br>Q3: 1.68 (0.62)<br>Q4: 1.97 (0.67)	OA: 2.51 (0.43)<br>Q1: 3.12 (0.57)<br>Q2: 2.83 (0.52)<br>Q3: 2.56 (0.77)<br>Q4: 1.38 (0.84)	OA, Q4<br>IF > STxF	OA, Q1-4<br>IF > IM	OA, Q1,Q2<br>STxM > IM	N.S.
Count non-bout	OA: 3.92 (0.45)<br>Q1: 3.59 (0.6)<br>Q2: 3.97 (0.54)<br>Q3: 3.28 (0.83)<br>Q4: 4.2 (0.93)	OA: 2.53 (0.39)<br>Q1: 2.22 (0.5)<br>Q2: 2.42 (0.46)<br>Q3: 3.01 (0.52)<br>Q4: 2.51 (0.56)	OA: 3.86 (0.42)<br>Q1: 3.69 (0.57)<br>Q2: 3.38 (0.53)<br>Q3: 4.26 (0.59)<br>Q4: 4.03 (0.64)	OA: 4.75 (0.42)<br>Q1: 4.76 (0.55)<br>Q2: 4.93 (0.5)<br>Q3: 4.87 (0.73)<br>Q4: 2.87 (0.88)	N.S.	OA, Q1,Q2<br>IM > IF	OA, Q1-3<br>STxM > IM	OA, Q2<br>STxM > STxF
Contraction frequency (Hz)	OA: 0.09 (0.01)<br>Q1: 0.09 (0.01)<br>Q2: 0.09 (0.01)<br>Q3: 0.08 (0.01)<br>Q4: 0.1 (0.01)	OA: 0.03 (0.01)<br>Q1: 0.03 (0.01)<br>Q2: 0.03 (0.01)<br>Q3: 0.03 (0.01)<br>Q4: 0.03 (0.01)	OA: 0.06 (0.01)<br>Q1: 0.07 (0.01)<br>Q2: 0.06 (0.01)<br>Q3: 0.04 (0.01)<br>Q4: 0.05 (0.01)	OA: 0.06 (0.01)<br>Q1: 0.07 (0.01)<br>Q2: 0.07 (0.01)<br>Q3: 0.05 (0.01)<br>Q4: 0.04 (0.01)	OA, Q1-4<br>IF > STxF	OA, Q1-4<br>IF > IM	OA, Q1,Q2<br>STxM > IM	N.S.
